# Reprogramming of Fatty Acid Metabolism via PPARα‐Orchestrated FADS2 in Keratinocytes Modulates Skin Inflammation in Psoriasis

**DOI:** 10.1002/advs.202417049

**Published:** 2025-08-29

**Authors:** Jiangluyi Cai, Xue Zhou, Yu Zhuang, Lian Cui, Rui Ma, Youdong Chen, Nan Yang, Qianyu Chen, Yuanyuan Wang, Peiyao Zhu, Lingling Yao, Qian Yu, Xiaomin She, Xuyang Zhou, Yuemeng Huang, Zengyang Yu, Xilin Zhang, Jiajing Lu, Yuling Shi, Chunyuan Guo

**Affiliations:** ^1^ Department of Dermatology, Shanghai Skin Disease Hospital, Institute of Psoriasis Tongji University School of Medicine Shanghai 200443 China; ^2^ Department of Dermatology, Shanghai Tenth People's Hospital, Institute of Psoriasis Tongji University School of Medicine Shanghai 200072 China

**Keywords:** FADS2, fatty acid, inflammation, keratinocytes, PPARα, psoriasis

## Abstract

Psoriasis is a chronic inflammatory skin disorder characterized by keratinocyte hyper‐proliferation and immune dysregulation. Recent evidence has implicated dysregulated polyunsaturated fatty acid (PUFA) metabolism in its pathogenesis. In this study, fatty acid desaturase 2 (FADS2), the rate‐limiting Δ6‐desaturase in PUFA biosynthesis, is identified as a central regulator of psoriatic inflammation. FADS2 expression is consistently reduced in keratinocytes from patients with psoriasis and in mouse models. Keratinocyte‐intrinsic *Fads2* knockdown exacerbates imiquimod‐induced psoriasis‐like dermatitis, which is marked by enhanced neutrophil recruitment and NF‐κB activation, whereas *Fads2* overexpression exerts protective effects and alleviates skin inflammation. In vitro, *FADS2* knockdown in keratinocytes enhances M5‐induced pro‐inflammatory cytokine production, whereas *FADS2* overexpression attenuates these effects. Lipidomic analysis reveals that impaired docosahexaenoic acid (DHA) biosynthesis is a key downstream consequence of FADS2 deficiency. Mechanistically, loss of FADS2 disrupts DHA biosynthesis, thus promoting an inflammatory response accompanied by increased NF‐κB phosphorylation in keratinocytes to attract neutrophils. Furthermore, PPARα is identified as an upstream transcriptional activator of FADS2, and pharmacological activation of PPARα alleviates psoriatic inflammation in a FADS2‐dependent manner. Together, these findings uncover a PPARα‐FADS2‐DHA‐NF‐κB axis that links lipid metabolism to immune regulation in psoriasis, highlighting a potential therapeutic strategy for restoring cutaneous immune homeostasis.

## Graphical abstract

This study identifies fatty acid desaturase 2 (FADS2) as a key regulator linking polyunsaturated fatty acid (PUFA) metabolism to psoriatic inflammation. FADS2 deficiency impairs docosahexaenoic acid (DHA) biosynthesis, enhances NF‐κB signaling, and promotes neutrophil‐driven skin inflammation. PPARα transcriptionally activates FADS2, and its activation alleviates inflammation in a FADS2‐dependent manner, highlighting a therapeutic axis to restore immune homeostasis in psoriasis.

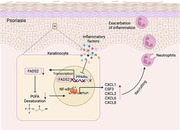

## 1 Introduction

Psoriasis is a chronic inflammatory skin disease characterized by excessive keratinocyte proliferation, abnormal differentiation, and immune cell infiltration.^[^ ^1^ ^]^ Driven primarily by the interleukin (IL)‐23/ T helper cell 17 (Th17) axis, the pathogenesis of psoriasis involves complex interactions between immune cells, including neutrophils, dendritic cells, T cells, and keratinocytes, which contribute to the initiation and maintenance of inflammation.^[^ ^2^ ^]^ Psoriasis is often associated with metabolic disturbances, particularly fatty acids, and studies have shown a strong link between fatty acid profiles and disease severity.^[^ ^3^ ^]^ Research has demonstrated an abnormal fatty acid profile in patients with psoriasis, with a significant association between disease severity (as measured by the Psoriasis Area Severity Index [PASI]) and low circulating levels of omega‐3 (n‐3) polyunsaturated fatty acids (PUFAs) and docosahexaenoic acid (DHA).^[^ ^4^ ^]^ The relationship between PUFAs and psoriasis severity exhibits both cross‐sectional and longitudinal differences between sexes. In females, the levels of n‐3 PUFAs, including eicosapentaenoic acid (EPA), DHA, and total n‐3 PUFAs, were inversely correlated with PASI and body surface area scores. Concentrations of dihomo‐γ‐linolenic acid were prospectively linked to an increase in PASI scores, whereas DHA was associated with a higher likelihood of achieving PASI 75 and PASI 90 improvements.^[^ ^3^ ^]^ These findings suggest that the dysregulation of PUFAs may play a crucial role in the pathogenesis of psoriasis. However, the exact mechanisms linking PUFA to inflammatory pathways in psoriasis remain unclear.

**Figure 1:**
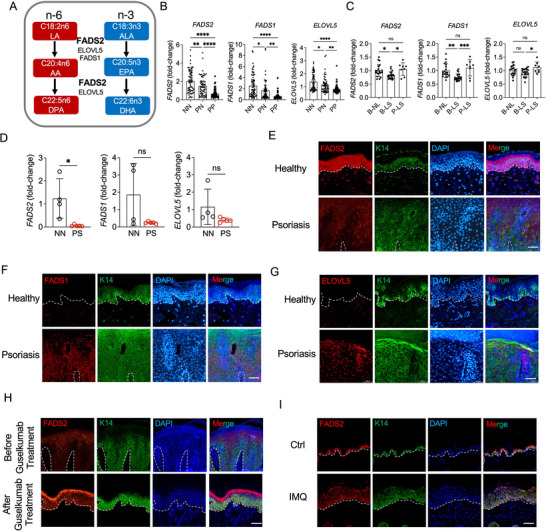
FADS2 is downregulated in keratinocytes of psoriatic lesions. A) Schematic illustration of the desaturation pathway of linoleic acid (omega‐6 [n‐6], red) and alpha‐linolenic acid (omega‐3 [n‐3], blue), catalyzed by key enzymes to generate long‐chain polyunsaturated fatty acids (LC‐PUFAs). B) Transcriptomic analysis of *FADS2, FADS1*, and *ELOVL5* expression in lesional (PP) and non‐lesional (PN) skin of psoriasis patients (n = 58) and in normal skin from healthy controls (NN, n = 64) based on the GEO dataset GSE13355. C) Transcriptomic analysis of *FADS2, FADS1*, and *ELOVL5* expression in baseline lesional (B‐LS) and non‐lesional (B‐NL) skin of psoriasis patients (n = 59), and in lesional skin after 12 weeks of guselkumab treatment (P‐LS), from GEO dataset GSE51440. D) RT‐qPCR validation of *FADS2, FADS1*, and *ELOVL5* expression in normal skin from healthy controls (NN) (n = 4) and lesional skin tissue from psoriasis patients (PS) (n = 5). E–G) Representative immunofluorescence images of FADS2 (E), FADS1 (F), and ELOVL5 (G) staining, along with the keratinocyte marker K14, in normal skin from healthy controls and lesional skin tissue from psoriasis patients. Dashed line indicates the border between the epidermis and dermis. H) Representative immunofluorescence images of FADS2 and K14 co‐staining in lesional skin tissue from psoriasis patients at baseline and after 12 weeks guselkumab treatment. I) Representative immunofluorescence images of FADS2 and K14 co‐staining in healthy skin from control mice (Ctrl) and imiquimod (IMQ)‐induced psoriatic skin lesions. Scale bar, 50 µm. Data are presented as mean ± SD. Statistical significance was determined by one‐way ANOVA (B,C) or unpaired two‐tailed Student's *t*‐test (D). ^*^ *P* < 0.05, ^**^ *P* < 0.01, ^***^ *P* < 0.001, ^****^ *P* < 0.0001; ns, not significant.

Δ6‐desaturase, also known as fatty acid desaturase 2 (FADS2), is an endoplasmic reticulum membrane‐bound protein that acts as a key enzyme in PUFA metabolism, as the initial rate‐limiting factor in the biosynthesis of long‐chain (≥C20) PUFAs (LC‐PUFAs). This biosynthesis pathway (**Figure** **1**A) is catalyzed by FADS2, FADS1, ELOVL5, and related enzymes through the desaturation and elongation of the omega‐6 (n‐6) PUFA linoleic acid (18:2 n‐6, LA) to arachidonic acid (20:4 n‐6, AA) and the n‐3α‐linolenic acid (18:3 n‐3, ALA) to EPA (20:5 n‐3) and DHA (22:6 n‐3).^[^ ^5^ ^]^ FADS2 plays a pivotal role in maintaining LC‐PUFA homeostasis ^[^ ^6^ ^]^ and its dysfunction has been linked to various chronic inflammatory diseases, such as type 2 diabetes, cardiovascular disease, obesity, Crohn's disease, and metabolic syndrome.^[^ ^7^, ^8^, ^9^, ^10^, ^11^ ^]^ Restoring FADS2 expression in human mesentery tissue enabled the endogenous synthesis of pro‐resolving lipid mediators from n‐3 fatty acids, led to a marked decrease in the infiltration of pro‐inflammatory macrophages, and significant suppression in the production of inflammatory cytokines and adipokines.^[7^^]^ The FADS2 genotype influences n‐6 desaturase activity and inflammatory processes in human adipose tissue. Genetic variations in FADS2 were further linked to the expression of IL‐1β and NF‐κB signaling pathway in subcutaneous adipose tissue following weight loss.^[^ ^12^ ^]^ It has also been reported that FADS2‐dependent desaturation can be activated by stimulator of interferon genes (STING) agonists, promoting metabolic alterations in PUFAs, thereby regulating antiviral responses and contributing to the resolution of STING‐associated inflammation through the inhibition of STING expression.^[^ ^13^ ^]^ In mouse models, FADS2 deficiency results in severe metabolic disorders, skin and intestinal ulcers, and reproductive issues, which can be alleviated by PUFA supplementation.^[^ ^14^, ^15^ ^]^ These findings underscore the significance of FADS2 in modulating inflammation via PUFA desaturation.

Given that previous studies have implicated PUFA in the pathogenesis of psoriasis,^[^ ^7^, ^16^, ^17^, ^18^ ^]^ and our previous transcriptomic analysis revealed dysregulation of fatty acid metabolism pathways in psoriasis, with markedly reduced FADS2 expression in psoriatic lesions,^[^ ^19^ ^]^ we hypothesized that FADS2 might play a protective role in psoriasis. However, its specific functions and regulatory mechanisms in psoriasis remain unclear. Considering the pivotal role of keratinocytes in psoriasis pathogenesis,^[^ ^20^ ^]^ we systematically investigated the expression pattern, functional significance, and regulatory mechanism of FADS2 in psoriatic keratinocytes using clinical samples as well as in vitro and in vivo models. We found significantly reduced FADS2 expression in psoriatic keratinocytes. Functional analyses revealed that FADS2 deficiency in keratinocytes enhanced inflammatory responses by activating the NF‐κB pathway to recruit neutrophils to the psoriasis lesions, likely through disruption of the n‐3 fatty acids desaturation pathway involved in DHA biosynthesis. Furthermore, we identified PPARα as a key upstream regulator of FADS2 in keratinocytes, and restoration of the PPARα‐FADS2 axis effectively ameliorated psoriatic inflammation both in vitro and in vivo. These findings identify a keratinocyte‐intrinsic mechanism by which FADS2 restrains psoriatic inflammation, highlighting the PPARα‐FADS2 axis as a potential therapeutic target for psoriasis.

## 2 Result

### 2.1 FADS2 is Downregulated in Keratinocytes of Psoriasis Lesions

PUFAs are essential components of skin structure and function, contributing to membrane integrity and the regulation of inflammatory responses.^[^ ^16^ ^]^ To investigate the involvement of PUFA biosynthesis in psoriasis, we examined the expression of key enzymes responsible for PUFAs synthesis (Figure 1A), including the desaturases *FADS1* and *FADS2* and the elongase *ELOVL5*, using publicly available microarray datasets (accession no. GSE13355 and GSE51440). The analysis revealed that all three enzymes were significantly downregulated in the lesional skin of psoriatic patients compared to the non‐lesional or healthy skin (Figure 1B). Importantly, the expression of *FADS1, FADS2*, and *ELOVL5* was restored by treatment with guselkumab, an IL‐23 inhibitor (Figure 1C). Among these enzymes, FADS2 showed the most consistent and robust down‐regulation. This finding was validated by reverse transcription quantitative polymerase chain reaction (RT‐qPCR), which showed a significant decrease in *FADS2* mRNA levels in psoriatic lesional skin compared to that in healthy skin (Figure 1D). Immunofluorescence staining further confirmed a marked reduction in FADS2 protein levels in psoriatic lesional skin, especially in keratinocytes (Keratin 14 [K14]‐positive cells), whereas FADS1 and ELOVL5 were upregulated at the protein level in the epidermis of skin lesions (Figure 1E–G). Consistently, FADS2 expression was restored following guselkumab treatment (Figure 1H). In the imiquimod (IMQ)‐induced psoriasis‐like mouse model, FADS2 expression in keratinocytes was significantly reduced in skin lesions and continued to decrease as the disease progressed (Figure 1I; Figure S1A, Supporting Information). Taken together, these findings demonstrate that FADS2 is consistently and specifically downregulated in the keratinocytes of both human psoriatic lesions and murine psoriasis‐like skin, suggesting a potential involvement of FADS2 dysregulation in the pathogenesis of psoriasis.

### 2.2 Silencing *Fads2* Aggravates Skin Inflammation and Enhances Neutrophil Infiltration in IMQ‐Induced Psoriasis‐Like Dermatitis

FADS2 modulates the inflammatory response by influencing metabolic pathways.^[^ ^13^ ^]^ Restoration of FADS2 expression has been shown to reduce pro‐inflammatory macrophage infiltration and suppress inflammatory mediators in diseases such as Crohn's disease, highlighting its potential therapeutic relevance.^[7^^]^

**Figure 2:**
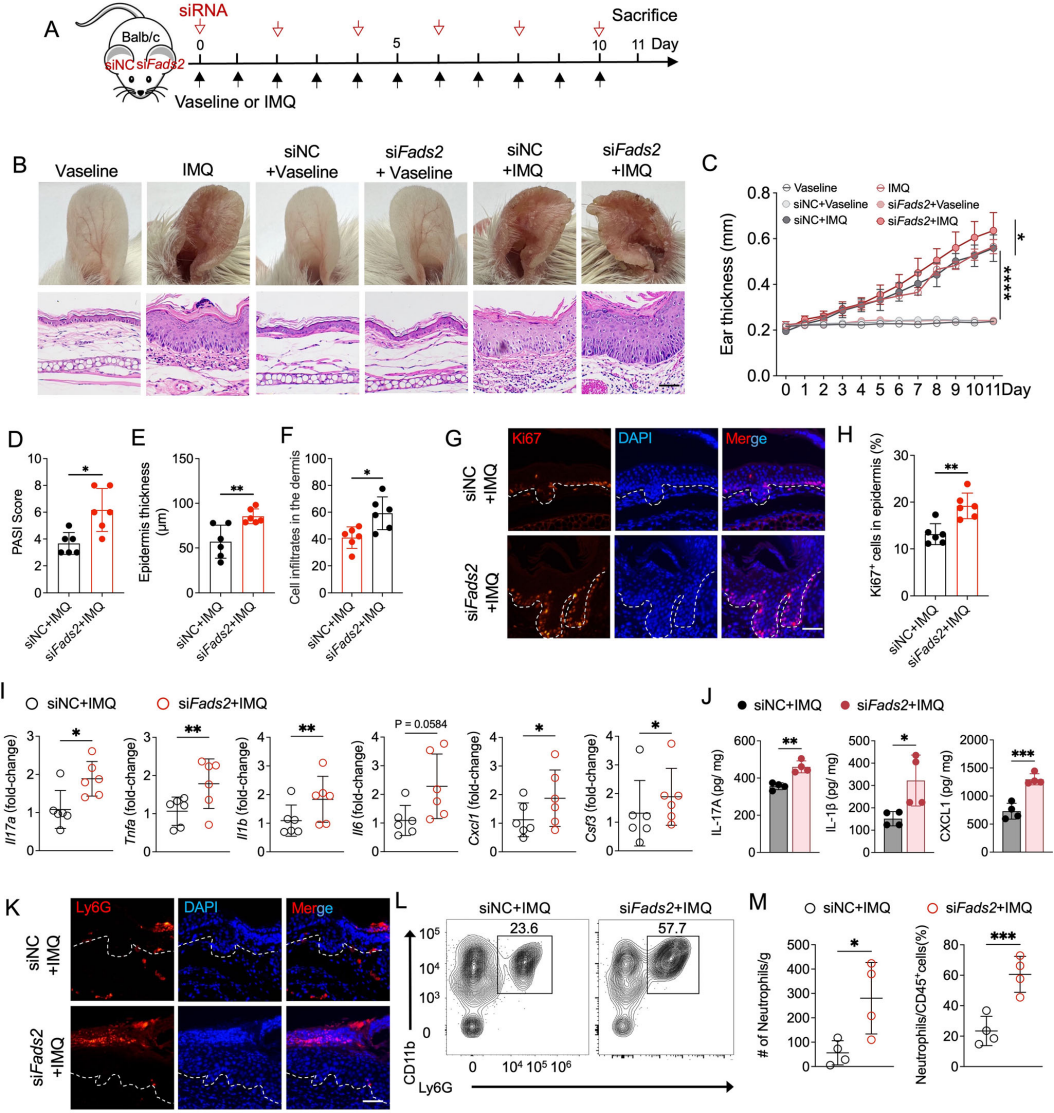
Silencing *Fads2* aggravates IMQ‐induced psoriatic skin inflammation and enhances neutrophil infiltration. A) Schematic diagram of strategy for the application of *Fads2* siRNA or control siRNA with IMQ or Vaseline treatment. B,C) Representative phenotypic images and hematoxylin‐eosin (H&E) staining image (B), and ear thickness of the indicated time points (C) following treatment with *Fads2* siRNA or control siRNA after Vaseline (n = 5) or IMQ (n = 6) application, as well as Vaseline or IMQ treatment alone (n = 4) for 11 days. D–F) Quantification of psoriasis area and severity index (PASI) scores (D), epidermal thickness (E), and dermal immune cell infiltration (F) in mice after 11 consecutive days of IMQ treatment with *Fads2* siRNA or control siRNA application (n = 6). G,H) Representative immunofluorescence images of Ki67 staining (G) and quantitation of Ki67^+^ epidermal cells (H) in IMQ‐induced skin lesions treated with *Fads2* siRNA or control siRNA (n = 6). I) RT‐qPCR analysis of the indicated genes in the skin lesions after 11 days of IMQ treatment with *Fads2* siRNA or control siRNA application (n = 6). J) ELISA quantification of IL‐17A, IL‐1β, and CXCL1 protein levels in the skin lesions following 11 days IMQ treatment with *Fads2* siRNA or control siRNA application (n = 4). K) Representative immunofluorescence images of Ly6G (a neutrophil marker) staining in IMQ‐induced skin lesions treated with *Fads2* siRNA or control siRNA. L,M) Representative flow cytometry plot (L) and quantification (M) of neutrophils in IMQ‐induced skin lesions treated with *Fads2* siRNA or control siRNA (n = 4). Scale bar, 50 µm. Data are presented as mean ± SD. Statistical analysis was determined by two‐way ANOVA (C) or paired two‐tailed Student's t test (D‐F,H‐J,M). ^*^ *P* < 0.05, ^**^ *P* < 0.01, ^***^ *P* < 0.001, ^****^ *P* < 0.0001.

To investigate the functional role of FADS2 in psoriasis, we used a mouse ear model of IMQ‐induced psoriasis‐like dermatitis. *Fads2*‐specific siRNA or control siRNA was administered every other day after IMQ application (**Figure** **2**A). Successful knockdown of *Fads2* in mouse skin was confirmed by RT‐qPCR and immunofluorescence staining (Figure S1B,C, Supporting Information). Mice treated with *Fads2* siRNA exhibited markedly aggravated IMQ‐induced skin inflammation, as evidenced by increased ear thickness, elevated PASI scores, pronounced epidermal hyperplasia, and enhanced dermal inflammatory cell infiltration (Figure 2B–F). Additionally, the proportion of Ki67^+^ cells, which indicates cell proliferation, was significantly elevated in the epidermis of IMQ‐treated mice with *Fads2* knockdown (Figure 2G,H). Furthermore, inflammatory cytokines relevant to psoriasis pathogenesis, including *Il17a*, *Tnfa*, and *Il1b*, as well as neutrophil‐attracting chemokines, such as *Cxcl1* and *Csf3*, were significantly upregulated at the mRNA level in *Fads2*‐silenced skin lesions compared to controls (Figure 2I). Consistently, at the protein level, increased expression of IL‐17A, IL‐1β, and CXCL1 was also observed following *Fads2* knockdown (Figure 2J). Given the enhanced expression of neutrophil‐attracting chemokines, we hypothesized that FADS2 deficiency contributes to enhanced neutrophil recruitment, a well‐known feature of psoriasis pathology marked by Munro's microabscesses in the stratum corneum.^[^ ^21^, ^22^ ^]^ Immunofluorescence staining revealed prominent accumulation of neutrophils in the stratum corneum of *Fads2*‐silenced skin lesions (Figure 2K). Flow cytometry further confirmed a substantial increase in both the number and proportion of neutrophils in *Fads2*‐silenced skin lesions (Figure 2L,M).

Collectively, these results demonstrated that silencing *Fads2* amplified psoriasis‐like skin inflammation and promoted neutrophil infiltration, thereby underscoring FADS2 as a critical negative regulator of psoriatic inflammation.

### 2.3 *FADS2* Silencing Promotes Inflammatory Response in Psoriatic Keratinocytes Through NF‐κB Activation

**Figure 3:**
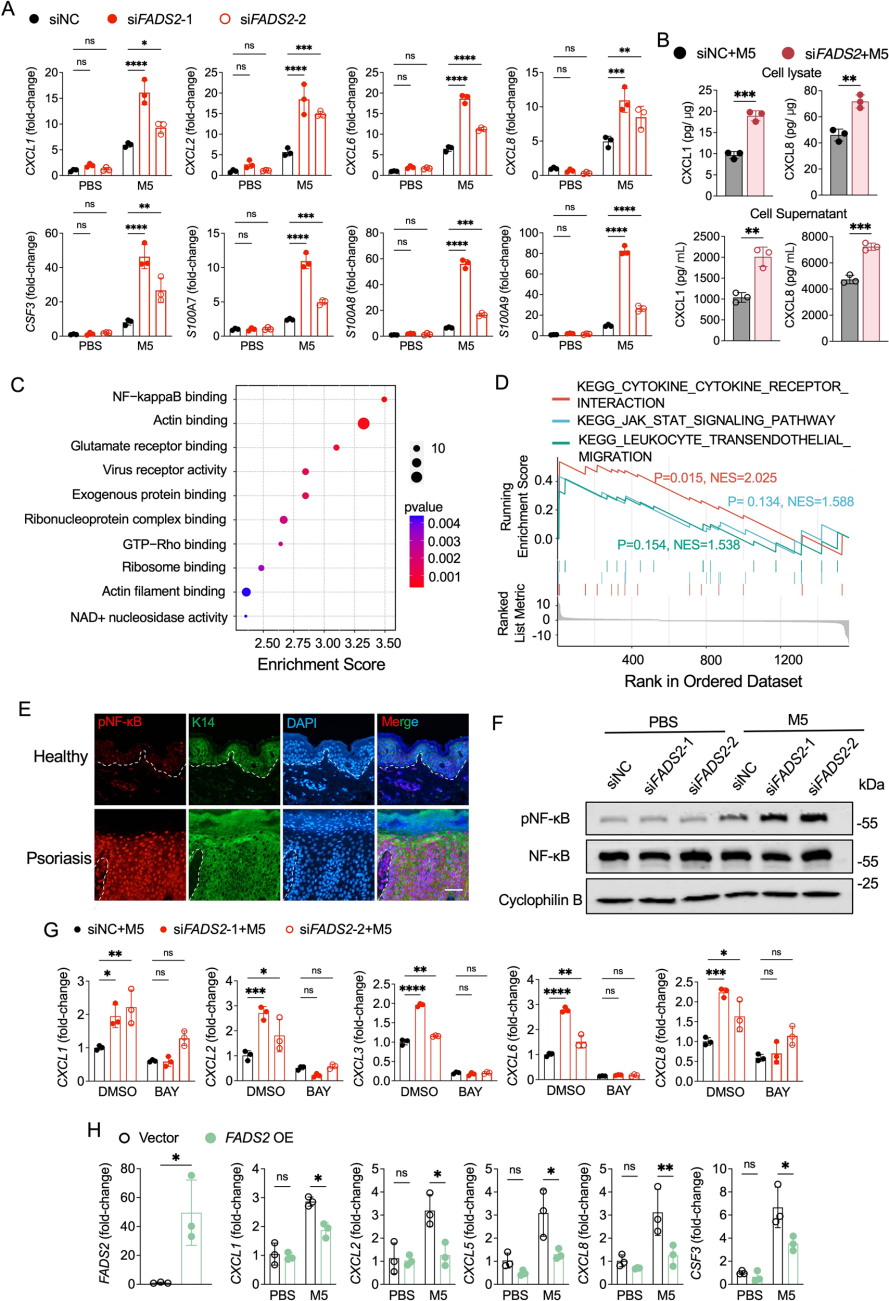
FADS2 modulates psoriatic inflammation in keratinocytes through NF‐κB activation. A) RT‐qPCR analysis of the indicated genes in HaCaT cells transfected with *FADS2* siRNA (si*FADS2*) or control siRNA (siNC) for 24 h, followed by stimulation with PBS or a cytokine cocktail (M5) for 12 h (n = 3). B) ELISA quantification of CXCL1 and CXCL8 protein levels in both cell lysates and supernatants of HaCaT cells treated as (A) (n = 3). C) Top 10 enriched Gene Ontology (GO) molecular function terms from RNA‐sequencing (RNA‐seq) analysis of differential expression genes (DEGs) in *FADS2*‐silenced (si*FADS2*) versus control (siNC) HaCaT cells after M5 stimulation. DEGs were defined by |fold change| >1.5 & adjusted *P*<0.05. D) Gene set enrichment analysis (GSEA) of Kyoto Encyclopedia of Genes and Genomes (KEGG) pathway enrichment from RNA‐seq results in *FADS2*‐silenced (si*FADS2*) versus control (siNC) HaCaT cells after M5 stimulation. E) Representative immunofluorescence images of phosphorylated NF‐κB p65 (pNF‐κB) and K14 co‐staining in normal skin from healthy controls and lesional skin tissue from psoriasis patients. F) Immunoblotting of pNF‐κB and total‐NF‐κB p65 (NF‐κB) in HaCaT cells transfected with si*FADS2* or siNC for 24 h and stimulated with PBS or M5 for 1 h. G) RT‐qPCR analysis of the indicated genes in HaCaT cells transfected with si*FADS2* or siNC and stimulated with M5 after BAY 11–7082 or DMSO pretreatment (n = 3). H) RT‐qPCR analysis of *FADS2* and the indicated genes in HaCaT cells transfected with *FADS2* overexpression plasmids (*FADS2* OE) or empty vector for 48 h followed by PBS or M5 treatment for 10 h (n = 3). Scale bar, 50 µm. Data are presented as mean ± SD. Statistical significance was determined by two‐way ANOVA (A,G,H) or unpaired two‐tailed Student's *t*‐test (B, H). ^*^ *P* < 0.05, ^**^ *P* < 0.01, ^***^ *P* < 0.001, ^****^ *P* < 0.0001; ns, not significant.

Given the specific down‐regulation of FADS2 in psoriatic keratinocytes, we examined its role in keratinocyte‐driven inflammation. Using siRNA, *FADS2* was silenced in cultured human keratinocyte cell line—HaCaT cells (Figure S2A,B, Supporting Information). An in vitro psoriatic milieu was simulated by the M5 cytokine cocktail (IL‐17A, TNF‐α, IL‐1α, IL‐22, and oncostatin M).^[^ ^23^ ^]^ After M5 stimulation, *FADS2*‐silenced HaCaT cells displayed significantly increased mRNA expression of neutrophil chemoattractants (*CXCL1*, *CXCL2*, *CXCL6*, *CXCL8*, and *CSF3*) compared to control cells, as well as the antimicrobial peptides *S100A7, S100A8*, and *S100A9* (**Figure** **3**A). Correspondingly, elevated protein levels of CXCL1 and CXCL8 were detected in both the cellular extracts and conditioned medium of *FADS2*‐silenced HaCaT cells in a psoriatic inflammatory environment (Figure 3B).

To elucidate the underlying mechanism, RNA sequencing (RNA‐seq) was performed on *FADS2*‐silenced HaCaT cells post‐M5 stimulation. Gene ontology (GO) enrichment analysis of differentially expressed genes (DEGs) identified “NF‐κB binding” as a top molecular function (Figure 3C), while the gene set enrichment analysis (GSEA) of kyoto encyclopedia of gand genomes (KEGG) pathway enrichment highlighted “cytokine‐cytokine receptor interaction” as a key pathway (Figure 3D). NF‐κB activation drives the transcription of pro‐inflammatory mediators and immune‐regulatory genes in psoriasis, thereby contributing to sustained skin inflammation.^[^ ^24^ ^]^ Our immunofluorescence staining validated the enhanced phosphorylation of NF‐κB p65 in psoriatic epidermis (Figure 3E), and *FADS2* knockdown increased NF‐κB p65 phosphorylation in M5‐stimulated HaCaT cells (Figure 3F; Figure S2C, Supporting Information). Treatment with the NF‐κB inhibitor BAY 11–7082 reversed the M5‐induced inflammatory phenotype exacerbated by *FADS2* knockdown, reinforcing the regulatory role of FADS2 via NF‐κB signaling in psoriatic inflammation (Figure 3G). Consistent with this, elevated NF‐κB phosphorylation was evident in the IMQ‐induced psoriasis‐like skin lesions after *Fads2* knockdown (Figure S2D, Supporting Information). Conversely, *FADS2* overexpression attenuated the upregulation of neutrophil‐attracting chemokines in M5‐stimulated HaCaT cells (Figure 3H).

Collectively, these findings support a role for FADS2 in restraining keratinocyte‐driven inflammatory responses through modulation of NF‐κB signaling.

### 2.4 Keratinocyte‐Intrinsic FADS2 Modulates Psoriatic Inflammation In Vivo

**Figure 4:**
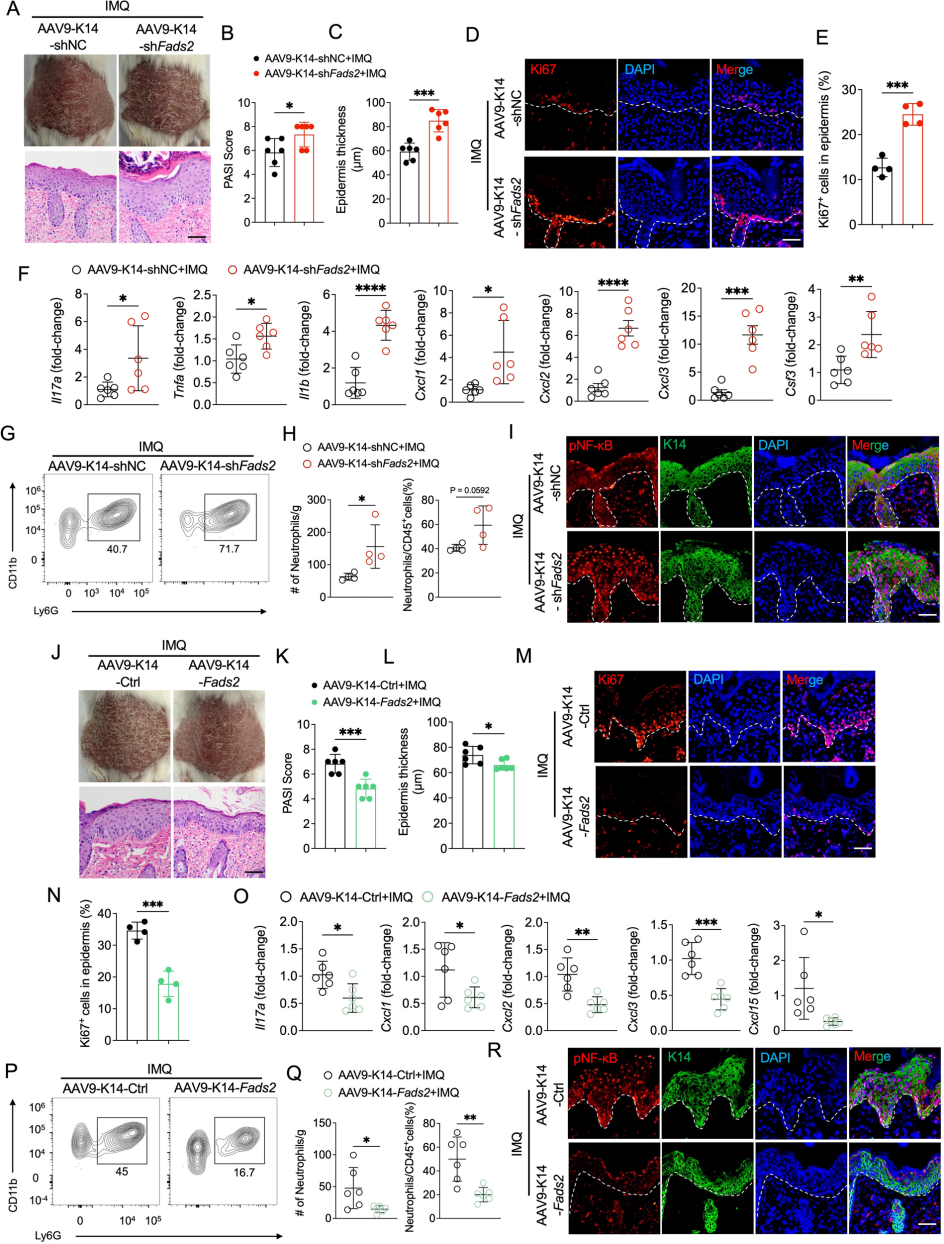
Keratinocyte‐specific modulation of FADS2 expression by AAV9‐K14 delivery system alters IMQ‐induced skin inflammation. A‐I) BALB/c mice were subjected to IMQ‐induced psoriasis‐like dermatitis on the dorsal skin three weeks after intradermal injection of AAV9‐K14‐sh*Fads2* or AAV9‐K14‐shNC. A) Representative phenotypic images and H&E staining images of IMQ‐induced skin lesions treated as (A‐I). B,C) Quantification of PASI scores (B) and epidermal thickness (C) of IMQ‐induced skin lesions treated as (A‐I) (n = 6). D,E) Representative immunofluorescence images of Ki67 staining (D) and quantitation of Ki67^+^ epidermal cells (E) in IMQ‐induced skin lesions treated as (A‐I) in the indicated locations (n = 4). F) RT‐qPCR analysis of the indicated genes in IMQ‐induced skin lesions treated as (A‐I) (n = 6). G,H) Representative flow cytometry plot (G) and quantification (H) of neutrophils in IMQ‐induced skin lesions treated as (A‐I) (n = 4). I) Representative immunofluorescence images of pNF‐κB and K14 co‐staining in IMQ‐induced skin lesions treated as (A‐I). J‐R) BALB/c mice were subjected to IMQ‐induced psoriasis‐like dermatitis on the dorsal skin three weeks after intradermal injection of AAV9‐K14‐flag‐*Fads2* (AAV9‐K14‐*Fads2*) or AAV9‐K14‐NC (AAV9‐K14‐Ctrl). J) Representative phenotypic images and H&E staining images of IMQ‐induced skin lesions treated as (J–R). K,L) Quantification of PASI scores (K), epidermal thickness (L) in IMQ‐induced skin lesions treated as (J–R) (n = 6). M,N) Representative immunofluorescence images of Ki67 staining (M) and quantitation of Ki67^+^ epidermal cells (N) in IMQ‐induced skin lesions treated as (J‐R) (n = 4). O) RT‐qPCR analysis of the indicated genes in IMQ‐induced skin lesions treated as (J–R) (n = 6). P,Q) Representative flow cytometry plot (P) and quantification (Q) of neutrophils in IMQ‐induced skin lesions treated as (J–R) (n = 6). R) Representative immunofluorescence images of pNF‐κB and K14 co‐staining in IMQ‐induced skin lesions treated as (J–R). Scale bar, 50 µm. Data are presented as mean ± SD. Statistical significance was determined by unpaired two‐tailed Student's *t*‐test. ^*^ *P* < 0.05, ^**^ *P* < 0.01, ^***^ *P* < 0.001, ^****^ *P* < 0.0001; ns, not significant.

To directly evaluate the role of keratinocyte‐intrinsic FADS2 in psoriatic inflammation in vivo, we used an AAV9 delivery system driven by the K14 promoter to specifically manipulate FADS2 expression in epidermal keratinocytes. Three weeks after the intradermal administration of AAV‐K14‐sh*Fads2*, a substantial reduction in FADS2 mRNA and protein levels was observed in the epidermis of both the ear and the dorsal skin of mice (Figures S3A,B and S4A,B, Supporting Information). Following IMQ application, mice with keratinocyte‐specific *Fads2* knockdown developed more severe psoriasis‐like skin inflammation in both the ear and the dorsal skin, as evidenced by elevated PASI scores and pronounced epidermal hyperplasia (**Figure** **4**A–C; Figure S4C–F, Supporting Information). Epidermal proliferation was also significantly elevated, as indicated by an increased proportion of Ki67^+^ cells (Figure 4D,E; Figure S4G,H, Supporting Information). Moreover, mice with keratinocyte‐specific *Fads2* knockdown exhibited increased mRNA levels of psoriasis‐associated inflammatory mediators, including *Il17a*, *Il1b*, and *Cxcl1*, in both the dorsal and ear skin (Figure 4F; Figure S4J, Supporting Information), and greater neutrophil infiltration was confirmed by flow cytometry (Figure 4G,H; Figure S4K,L, Supporting Information). Importantly, NF‐κB phosphorylation was significantly enhanced in IMQ‐induced skin lesions of keratinocyte‐specific *Fads2*‐konckdown mice (Figure 4I; Figure S4I, Supporting Information).

Conversely, epidermal overexpression of *Fads2* via AAV‐K14‐flag‐*Fads2* delivery led to a marked upregulation of both FADS2 mRNA and protein levels (Figure S3C,D, Supporting Information), and markedly alleviated IMQ‐induced psoriasis‐like inflammation (Figure 4J). These mice displayed reduced PASI scores, attenuated epidermal hyperplasia, fewer Ki67⁺ keratinocytes, suppressed expression of psoriatic inflammatory mediators, and decreased neutrophil infiltration (Figure 4K–Q). Moreover, NF‐κB phosphorylation was markedly reduced in IMQ‐induced lesions of keratinocyte‐specific *Fads2*‐overexpressing mice (Figure 4R).

Collectively, these results demonstrate that keratinocyte‐intrinsic FADS2 critically regulates psoriatic skin inflammation, accompanied by modulating NF‐κB activity and neutrophil recruitment.

### 2.5 Decreased FADS2 Disrupts PUFA Metabolism, Amplifying Psoriatic Inflammation in Keratinocytes

**Figure 5:**
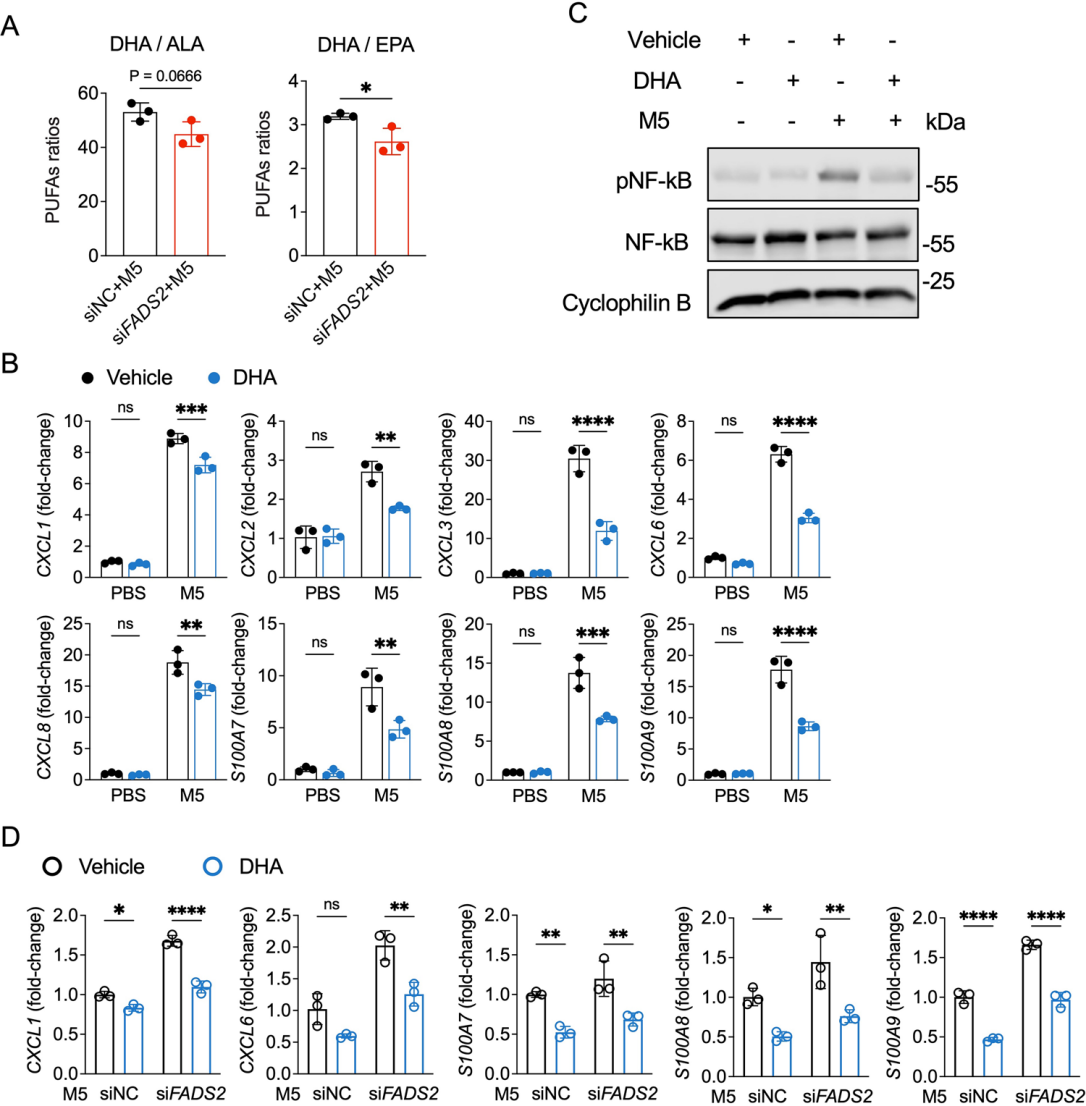
Decreased FADS2 disrupts PUFA metabolism that orchestrates psoriasiform inflammation in keratinocytes. A) PUFA ratios from LC‐MS/MS‐based lipidomic analysis showing desaturation indexes related to FADS2 in HaCaT cells transfected with *FADS2* siRNA and control siRNA after M5 stimulation (n = 3). B) RT‐qPCR analysis of the indicated genes in HaCaT cells treated with DHA or vehicle for 24 h and stimulated with PBS or M5 for 12 h (n = 3). C) Immunoblotting of pNF‐κB and total‐NF‐κB in HaCaT cells treated with DHA or vehicle for 24 h and stimulated with M5 for 1 h. D) RT‐qPCR analysis of the indicated genes in HaCaT cells transfected with si*FADS2* or siNC and stimulated with M5 after DHA or vehicle pretreatment (n = 3). Data are presented as mean ± SD. Statistical significance was determined by unpaired two‐tailed Student's t test (A) or two‐way ANOVA (B,D). ^*^ *P* < 0.05, ^**^ *P* < 0.01, ^***^ *P* < 0.001, ^****^ *P* < 0.0001; ns, not significant.

FADS2 is a key rate‐limiting enzyme in the biosynthesis of n‐6 and n‐3 PUFAs,^[^ ^25^ ^]^ including AA, EPA, and DHA. PUFAs serve as precursors of a range of lipid mediators with pro‐ and anti‐inflammatory properties, including prostaglandins, leukotrienes, resolvins, and protectins.^[^ ^26^ ^]^ Lipidomic profiling of epidermal tissues from IMQ‐induced psoriatic lesions revealed significant alterations in PUFA composition, including changes in the relative abundances of ALA, LA, EPA, and DHA, compared to the Vaseline‐treated controls (Figure S5A, Supporting Information). Similarly, the knockdown of *FADS2* in HaCaT cells under inflammatory conditions also led to a dysregulated PUFA profile (Figure S5B, Supporting Information). To assess FADS2 enzymatic activity more accurately, we calculated a desaturation index, defined as the ratio of downstream LC‐PUFA derivatives to their upstream precursors, among the FADS2‐regulated fatty acids. FADS2‐deficient cells showed reduced DHA: ALA and DHA: EPA ratios following M5 stimulation, indicating impaired FADS2‐mediated DHA biosynthesis in psoriatic keratinocytes (**Figure** **5**A).

Given the compromised DHA biosynthesis following *FADS2* knockdown, we explored the anti‐inflammatory effects of DHA on psoriatic keratinocytes. DHA supplementation significantly suppressed the expression of neutrophil‐attracting chemokines (*CXCL1, CXCL2, CXCL3, CXCL6*, and *CXCL8*) and antimicrobial peptides (*S100A7, S100A8*, and *S100A9*) and concomitantly decreased NF‐κB phosphorylation in M5‐stimulated keratinocytes (Figure 5B,C). Moreover, DHA treatment attenuated the enhanced inflammatory response observed in *FADS2*‐silenced HaCaT cells after M5 stimulation (Figure 5D). Taken together, these data suggested that FADS2 maintains PUFA metabolic homeostasis, particularly DHA synthesis, to prevent keratinocyte‐driven inflammation in psoriasis.

### 2.6 PPARα Acts as an Upstream Regulator of FADS2 in Epidermal Inflammation

**Figure 6:**
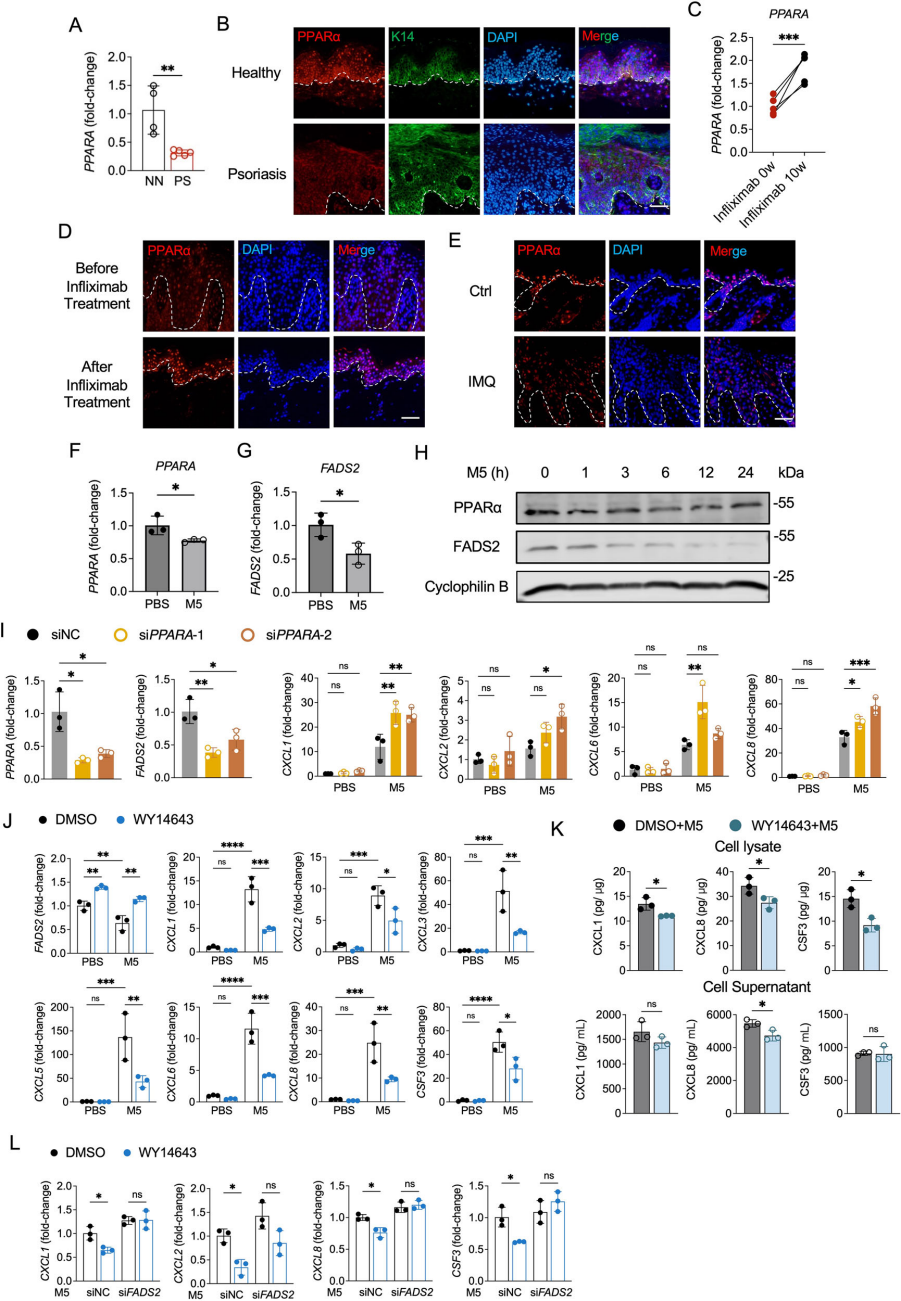
PPARα acts as an upstream positive regulator of FADS2 in psoriatic keratinocytes. A) RT‐qPCR analysis of *PPARA* expression in normal skin from healthy controls (NN, n = 4) and lesional skin tissue from psoriasis patients (PS, n = 5). B) Representative immunofluorescence images of PPARα and K14 co‐staining in normal skin from healthy controls and lesional skin tissue from psoriasis patients. C) RT‐qPCR analysis of *PPARA* in psoriatic lesional skin before and 10 weeks after infliximab treatment (n = 5). D) Representative images of PPARα immunofluorescence staining in lesional skin from psoriasis patients at baseline and week 10 following infliximab treatment. E) Representative immunofluorescence images of PPARα staining in healthy skin from control mice and skin lesions from IMQ‐induced psoriasis mouse model. F,G) RT‐qPCR analysis of *PPARA* (F) and *FADS2* (G) expression in HaCaT cells stimulated with M5 for 12 h (n = 3). H) Immunoblotting of PPARα and FADS2 in HaCaT cells stimulated with M5 cytokines for the indicated time. I) RT‐qPCR analysis of *PPARA*, *FADS2*, and indicated genes in HaCaT cells transfected with *PPARA* siRNA (si*PPARA*) and control siRNA (siNC) for 24 h, followed by PBS or M5 stimulation for 12 h (n = 3). J) RT‐qPCR analysis of *FADS2* and indicated genes in HaCaT cells pretreated with WY14643 or DMSO for 21 h, followed by PBS or M5 stimulation for 3 h (n = 3). K) ELISA quantification of CXCL1, CXCL8, and CSF3 protein levels in cell lysates and supernatants from HaCaT cells treated as in (J) (n = 3). L) RT‐qPCR analysis of the indicated genes in HaCaT cells transfected with si*FADS2* and siNC for 24 h, followed by M5 stimulation for 3 h before WY14643 or DMSO pretreatment (n = 3). Scale bar, 50 µm. Data are presented as mean ± SD. Statistical significance was determined by unpaired two‐tailed Student's t test (A,F,G,K), paired two‐tailed Student's t test (C), or one‐way ANOVA (I,J,L). ^*^ *P* < 0.05, ^**^ *P* < 0.01, ^***^ *P* < 0.001, ^****^ *P* < 0.0001; ns, not significant.

Previous studies have shown that FADS2 expression is positively regulated by the transcription factors PPARα and SREBP1.^[^ ^27^ ^]^ To identify the upstream regulators of FADS2 in psoriatic keratinocytes, we first analyzed the expression of *PPARA* and *SREBP1* using the publicly available dataset GSE13355 and our previously published RNA‐seq data.^[^ ^19^ ^]^ Notably, *PPARA* expression was reduced in psoriatic lesions, mirroring the downregulation of *FADS2*, while *SREBP1* expression remained unchanged or was slightly increased (Figure S6A–D, Supporting Information). These results suggest that PPARα, a nuclear receptor known to control lipid metabolism and fatty acid biosynthesis,^[^ ^28^, ^29^, ^30^, ^31^, ^32^ ^]^ is the more likely upstream regulator of FADS2 under psoriatic conditions. Upon ligand binding, the PPARα forms a heterodimer with retinoid X receptor α, which binds to peroxisome proliferator response elements within the FADS2 promoter, thereby modulating FADS2 transcription and the subsequent biosynthesis of LC‐PUFAs.^[^ ^27^, ^33^ ^]^ However, whether this regulatory axis operates under inflammatory conditions in keratinocytes, particularly in psoriasis, remains unclear. We found that mRNA and protein levels of PPARα were dramatically decreased in the lesional skin of psoriatic patients compared to healthy controls, with downregulation primarily localized to epidermal keratinocytes (**Figure** **6**A,B). Notably, treatment with infliximab, a TNF‐α inhibitor, restored PPARα expression in patient skin lesions (Figure 6C,D), aligning with the observed restoration of FADS2 expression after treatment (Figure 1H). Consistent findings were observed in IMQ‐induced murine psoriasis, where both PPARα and FADS2 expression were reduced in lesional epidermis compared to controls (Figure 6E and 1I). Similarly, both FADS2 and PPARα expression levels were significantly reduced in M5‐induced inflamed keratinocytes (Figure 6F–H).

To experimentally validate this regulatory relationship, HaCaT cells were transfected with *PPARA*‐targeting siRNA or treated with the PPARα agonist WY14643. As expected, *PPARA* knockdown led to reduced *FADS2* expression and significantly increased expression of *CXCL1* and *CXCL8* upon M5 stimulation (Figure 6I). Conversely, WY14643 treatment upregulated *FADS2* expression under both non‐inflammatory and inflammatory conditions and inhibited the M5‐induced expression of neutrophil chemoattractants (*CXCL1, CXCL2, CXCL3, CXCL5, CXCL6, CXCL8* and *CSF3*) (Figure 6J). Consistently, the cellular protein levels of CXCL1, CXCL8, and CSF3 were reduced in WY14643‐treated keratinocytes, along with a decrease in CXCL8 in the conditioned medium under psoriatic‐like conditions (Figure 6K).

To determine whether the anti‐inflammatory effect of PPARα activation is dependent on FADS2, we knocked down *FADS2*, followed by WY14643 treatment in M5‐stimulated HaCaT cells. The ability of WY14643 to suppress inflammatory mediators was abolished in FADS2‐deficient cells, indicating that FADS2 is a necessary effector downstream of PPARα in keratinocytes (Figure 6L).

Together, these findings indicate that PPARα transcriptionally regulates FADS2 and exerts anti‐inflammatory effects in keratinocytes through a PPARα‐FADS2 axis, which is critically impaired under psoriatic conditions.

### 2.7 PPARα Agonist WY14643 Alleviates IMQ‐Induced Psoriasis‐Like Skin Inflammation

**Figure 7:**
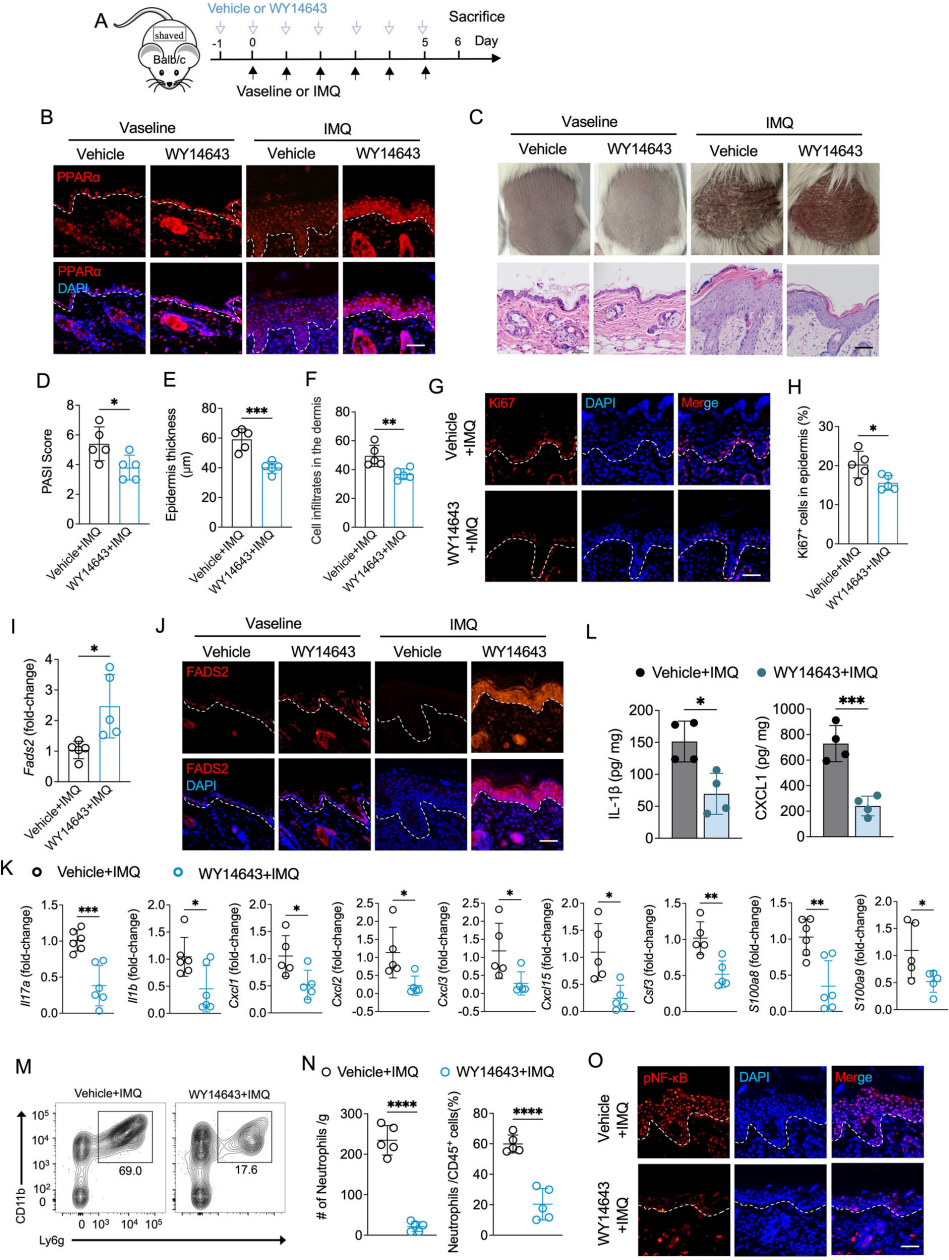
PPARα agonist WY14643 alleviates IMQ‐induced psoriasis‐like skin inflammation. A) Schematic diagram of experimental design involving topical application of WY14643 or vehicle in combination with IMQ or Vaseline treatment. B) Representative immunofluorescence images of PPARα staining in dorsal skin from mice treated with WY14643 or vehicle after IMQ or Vaseline treatment. C) Representative phenotypic images and H&E staining image of mouse dorsal skin treated with WY14643 or vehicle following IMQ or Vaseline treatment for 6 days. D–F) Quantification of PASI scores (D), epidermal thickness (E), and dermal cell infiltration (F) of mouse dorsal skin treated with WY14643 or vehicle after IMQ treatment for 6 days (n = 5). G,H) Representative immunofluorescence staining of Ki67 (G) and quantitation of Ki67^+^ epidermal cells (H) in IMQ‐induced skin lesions treated with WY14643 or vehicle in the indicated locations (n = 5). I) RT‐qPCR analysis of *Fads2* in IMQ‐induced skin lesions treated with WY14643 or vehicle (n = 5). J) Representative immunofluorescence images of FADS2 staining in mouse dorsal skin treated with WY14643 or vehicle after IMQ or Vaseline treatment for 6 days. K) RT‐qPCR analysis of the indicated genes in IMQ‐induced skin lesions treated with WY14643 or vehicle (n = 5‐6). L) ELISA quantification of IL‐1β and CXCL1 protein levels in IMQ‐induced skin lesions treated with WY14643 or vehicle (n = 4). M,N) Representative flow cytometry plot (M) and quantification (N) of neutrophils in IMQ‐induced skin lesions treated with WY14643 or vehicle (n = 5). O) Representative immunofluorescence images of pNF‐κB staining in IMQ‐induced skin lesions treated with WY14643 or vehicle. Scale bar, 50 µm. Data are presented as mean ± SD. Statistical significance was determined by unpaired two‐tailed Student's t test. ^*^ *P* < 0.05, ^**^ *P* < 0.01, ^***^ *P* < 0.001, ^****^ *P* < 0.0001; ns, not significant.

To investigate the role of PPARα in modulating psoriatic inflammation in vivo, we topically applied WY14643 or vehicle to the dorsal skin of mice treated with IMQ or vaseline (**Figure** **7**A). Immunofluorescence staining confirmed the upregulation of PPARα expression in epidermal keratinocytes following WY14643 treatment (Figure 7B). Compared to vehicle controls, IMQ‐treated mice receiving WY14643 exhibited significantly alleviated psoriasis‐like skin symptoms, including lower PASI scores, decreased epidermal hyperplasia, and reduced dermal inflammatory cell infiltration (Figure 7C–F). A modest decrease in Ki67⁺ proliferating keratinocytes was also observed (Figure 7G,H). Furthermore, PPARα activation by WY14643 restored FADS2 expression in epidermal keratinocytes in both non‐inflammatory and inflammatory states (Figure 7I,J). WY14643 treatment significantly downregulated psoriasis‐associated inflammatory cytokines and chemokines (*Il17a, Il1b, Cxcl1, Cxcl2, Cxcl3, Cxcl15*, and *Csf3*) as well as the antimicrobial peptides *S100a8* and *S100a9* in IMQ‐induced skin lesions at the transcriptional level (Figure 7K). At the protein level, IL‐1β and CXCL1 were also markedly decreased (Figure 7L). Flow cytometry revealed a reduction in neutrophil infiltration in IMQ‐induced skin lesions treated with WY14643 (Figure 7M,N), and immunofluorescence staining showed decreased NF‐κB phosphorylation in IMQ‐induced skin lesions upon PPARα activation (Figure 7O).

Collectively, these results demonstrate that topical activation of PPARα by WY14643 effectively alleviates psoriatic skin inflammation in vivo, accompanied by restoring FADS2 expression and suppressing neutrophil recruitment.

### 2.8 FADS2 Silencing Abolishes PPARα‐Mediated Amelioration of Psoriatic Skin Inflammation

**Figure 8:**
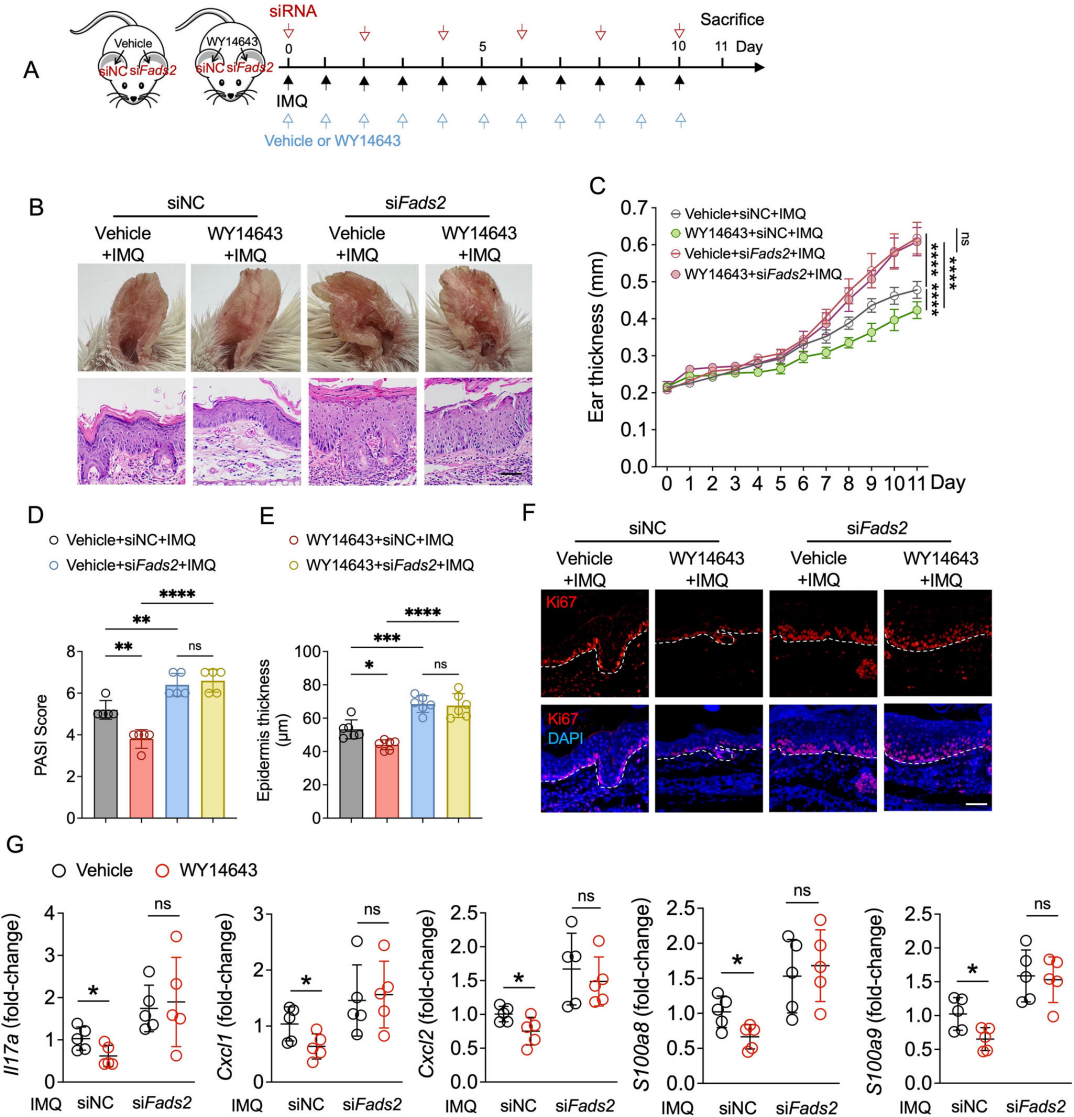
*Fads2* knockdown abrogates the anti‐inflammatory effects of the PPARα agonist WY14643 in IMQ‐induced psoriasis‐like inflammation. A) Schematic diagram of experimental design involving topical administration of WY14643 or vehicle followed by IMQ treatment, prior to si*Fads2* or siNC application on mouse ear for 11 days. B,C) Representative phenotypic images and H&E staining images (B), ear thickness of the indicated time points (C) of ear skin lesion treated as (A). D,E) Quantification of PASI scores (D) (n = 5), epidermal thickness (E) (n = 6) of ear skin lesions treated as (A). F) Representative immunofluorescence images of Ki67 staining in IMQ‐induced skin lesion treated as (A). G) RT‐qPCR analysis of the indicated genes in IMQ‐induced skin lesion treated as (A) (n = 5). Scale bar, 50 µm. Data are presented as mean ± SD. Statistical significance was determined by two‐way ANOVA (C), one‐way ANOVA (D,E), or unpaired two‐tailed Student's *t*‐test (G). ^*^ *P* < 0.05, ^**^ *P* < 0.01, ^***^ *P* < 0.001, ^****^ *P* < 0.0001; ns, not significant.

To assess whether FADS2 is required for PPARα‐mediated suppression of psoriatic inflammation in vivo, we knocked down *Fads2* using siRNA and administered WY14643 in IMQ‐induced psoriasis‐like skin lesions (**Figure** **8**A). Immunofluorescence staining confirmed that WY14643 did not restore FADS2 expression after silencing (Figure S7A,B, Supporting Information). WY14643 treatment failed to alleviate the exacerbated psoriatic phenotype in *Fads2*‐knockdown mice (Figure 8B). In contrast to the reduction observed in the control (siNC) group, no significant changes in ear thickness, PASI scores, or epidermal hyperplasia were noted following WY14643 treatment in the *Fads2* knockdown group (Figure 8C–E). Ki67⁺ keratinocyte frequency was only reduced in the siNC group, not in the *Fads2*‐knockdown skin (Figure 8F). Similarly, WY14643 significantly downregulated the expression of *Il17a*, *Cxcl1*, *Cxcl2*, *S100a8*, and *S100a9* in IMQ‐induced skin lesions in the siNC group; however, this inhibitory effect was diminished in the *Fads2* knockdown group (Figure 8G).

Collectively, these results establish FADS2 as an essential downstream effector for PPARα‐mediated immune modulation in psoriasis. The loss of therapeutic efficacy of WY14643 in FADS2‐deficient mice strongly supports a mechanistic link between PPARα activation, FADS2 expression, and the resolution of psoriatic inflammation.

## 3 Discussion

**Figure 9:**
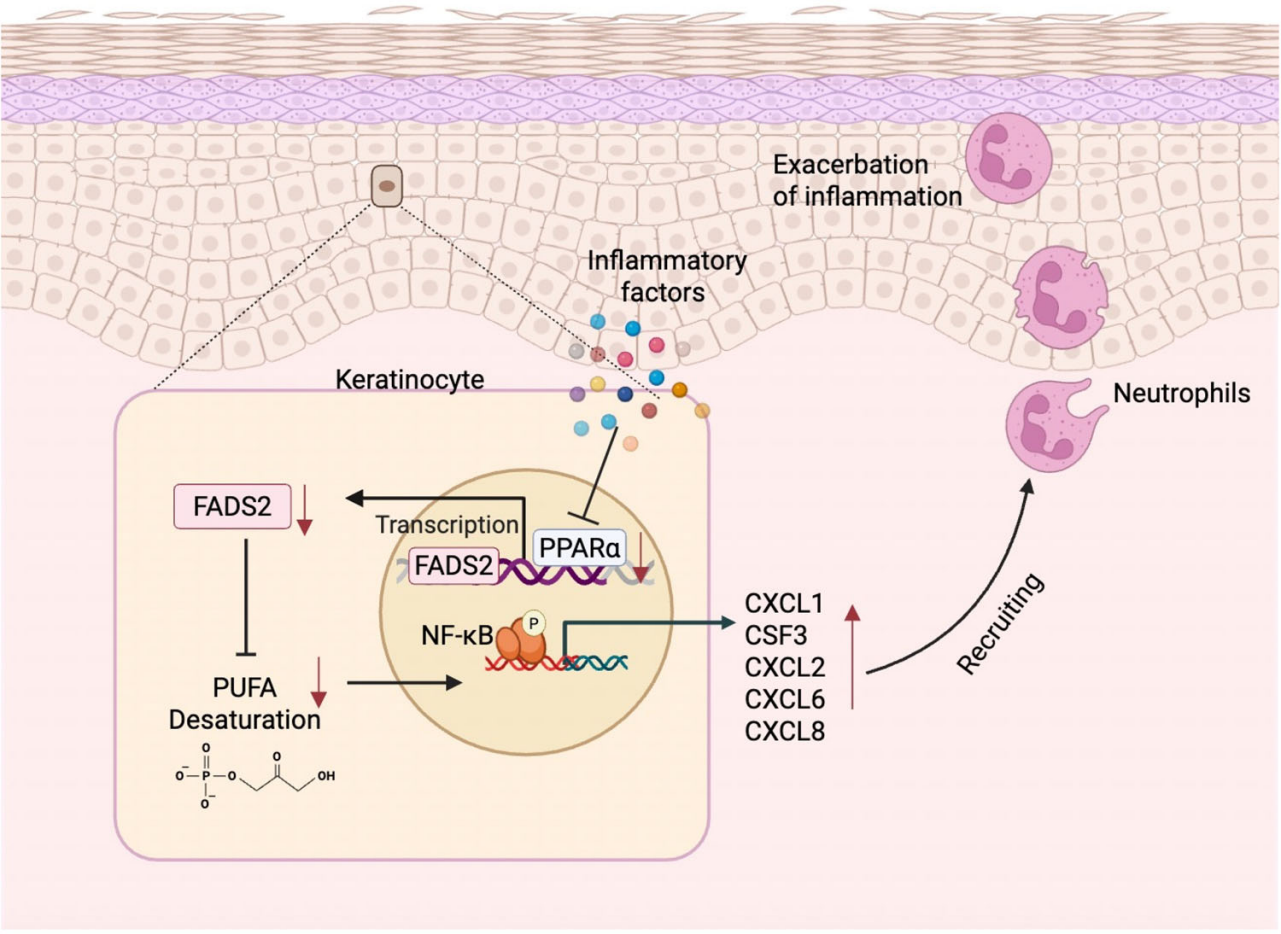
The PPARα‐FADS2‐PUFA axis regulates psoriasiform inflammation in keratinocytes. PPARα positively regulates FADS2 expression and thus promotes the desaturation of PUFAs. In psoriatic keratinocytes, reduced PPARα expression leads to downregulation of FADS2, resulting in decreased synthesis of DHA. This reduction enhances NF‐κB phosphorylation and subsequently upregulates psoriasis‐associated inflammatory cytokines and chemokines. These chemokines further promote neutrophil recruitment into skin lesions, aggravating psoriasis‐like inflammation. This figure was created with BioRender.com. Reproduced with permission. Copyright 2025, https://BioRender.com/rnyxsoi.

Psoriasis is a multifactorial, chronic inflammatory skin disorder characterized by dysregulated immune responses and aberrant keratinocyte behavior. Among various contributing factors, lipid metabolic reprogramming, particularly PUFA metabolism, has emerged as a pivotal factor in disease progression.^[^ ^16^ ^]^ In this study, we identified FADS2 as a key metabolic regulator of inflammation in psoriasis. Specifically, we demonstrate that FADS2 deficiency in keratinocytes disrupts PUFA homeostasis, enhances NF‐κB signaling, and promotes the production of neutrophil‐attracting chemokines, collectively aggravating cutaneous inflammation. Furthermore, we show that PPARα acts as an upstream positive regulator of FADS2, and that its pharmacologic activation alleviates psoriatic inflammation both in vitro and in vivo in a FADS2‐dependent manner. These findings reveal a functional PPARα–FADS2 axis that governs inflammatory signaling and lipid metabolism in keratinocytes and highlight a novel therapeutic target for psoriasis (**Figure** **9**).

Given the importance of PUFAs in regulating skin homeostasis, increasing attention has been paid to the upstream metabolic enzymes responsible for desaturation and elongation, particularly FADS2, FADS1, and ELOVL5.^[^ ^34^ ^]^ Clinical studies have linked reduced expression of FADS2 and ELOVL5 with an increased risk of atopic dermatitis, showing a significant correlation between lower gene expression and decreased plasma levels of n‐6 PUFAs in children.^[^ ^35^ ^]^ In psoriasis, transcriptomic analyses have consistently revealed the downregulation of lipid metabolism‐related genes, including FADS1 and FADS2, in lesional skin compared with healthy controls. Notably, the microarray dataset available for patients with psoriasis and IMQ‐induced mouse models both demonstrated significant reductions in FADS1, FADS2, and ELOVL5 expression in psoriatic skin lesions, indicating a conserved defect in PUFA biosynthesis in psoriatic inflammation.^[^ ^36^, ^37^ ^]^ In our study, we performed an integrative analysis combining public transcriptomic datasets, RT‐qPCR, and immunofluorescence staining to assess both the mRNA and protein expression levels of key PUFA biosynthetic enzymes (FADS2, FADS1, and ELOVL5) in psoriatic skin. Among these, only FADS2 exhibited consistent downregulation at both mRNA and protein levels in lesional skin, particularly in keratinocytes, corroborating previous findings on the dysregulation of lipid metabolism in psoriasis. While transcriptomic datasets revealed significant reductions in *FADS1* and *ELOVL5* mRNA levels in lesional skin compared to non‐lesional or healthy skin, RT‐qPCR results in our clinical samples demonstrated a similar trend without reaching statistical significance, likely due to variations among clinical samples and limited sample size. Notably, despite reduced mRNA levels, FADS1 and ELOVL5 protein levels were elevated in psoriatic epidermis, suggesting a possible compensatory upregulation at the post‐transcriptional level. Functionally, our in vivo experiments showed that keratinocyte‐specific *Fads2* knockdown in the IMQ‐induced psoriasis mouse model aggravated skin inflammation, increased keratinocyte proliferation, elevated the levels of inflammatory factors and chemokines, and promoted neutrophil infiltration. These results align with a previous study demonstrating severe inflammatory phenotypes in global FADS2‐deficient mice, including skin involvement.^[^ ^15^ ^]^ As the first and rate‐limiting Δ6‐desaturase, FADS2 governs the initial step in both the n‐3 and n‐6 LC‐PUFA biosynthesis pathways, positioning it as a central metabolic regulator. In contrast, FADS1 functions downstream as a Δ5‐desaturase, while ELOVL5 mediates the elongation of C18 to C20 fatty acids, providing substrates for both FADS2 and FADS1. Disruption of FADS2 leads to a broad impairment in PUFA desaturation capacity and has been implicated in multiple inflammatory diseases, such as inflammatory bowel disease.^[7^^]^ Altered FADS1 activity is associated with an imbalance in lipid mediators‐specially increased leukotriene production and reduced resolvin synthesis‐contributing to the pathogenesis of inflammatory disorders and cancer.^[^ ^38^ ^]^ ELOVL5 upregulation, particularly in immune and tumor cells, has been linked to enhanced PUFA flux, dysregulated lipid mediator profiles, susceptibility to inflammation, and ferroptosis.^[^ ^39^ ^]^ Although FADS1 and ELOVL5 share roles with FADS2 in PUFA biosynthesis, their upregulation may partially compensate for impaired lipid metabolism under inflammatory conditions.^[^ ^40^ ^]^ However, such compensation appears insufficient as inflammation and keratinocyte hyperactivation persist, underscoring the non‐redundant role of FADS2 in maintaining the epidermal lipid balance and immunological homeostasis.^[^ ^8^, ^15^ ^]^ This is further supported by knockout studies showing FADS2 deficiency causes severe systemic phenotypes, including hepatic steatosis, infertility, and cutaneous inflammation, which is not observed in FADS1 or ELOVL family knockout.^[^ ^34^ ^]^ These findings highlight that while FADS1 and ELOVL5 are essential components of the PUFA metabolic network, FADS2 uniquely serves as a metabolic gatekeeper, coordinating both PUFA biosynthesis and immunometabolic signaling.

Keratinocytes contribute substantially to the pathology of psoriasis by initiating and sustaining inflammation through abnormal differentiation, excessive proliferation, and overproduction of inflammatory mediators.^[^ ^20^ ^]^ Upon activation, they undergo hyperproliferation and secrete pro‐inflammatory mediators, such as CXCL1, CXCL2, CXCL8, and CCL20, which promote the recruitment of neutrophils, Th17 cells, and dendritic cells.^[^ ^41^ ^]^ Among these, the keratinocyte‐neutrophil axis plays a pivotal role in amplifying psoriatic inflammation.^[^ ^42^ ^]^ Keratinocytes secrete chemokines, such as CXCL1, CXCL8, and CSF3, which promote neutrophil migration into the lesional epidermis, leading to epidermal neutrophil infiltration and the formation of Munro's microabscesses. Neutrophils, upon infiltration, release cytokines (e.g., IL‐1β, IL‐6) and form neutrophil extracellular traps, which further activate keratinocytes, establishing a self‐amplifying inflammatory loop.^[^ ^43^, ^44^ ^]^ In this study, *FADS2* silencing in keratinocytes significantly upregulated the expression of neutrophil chemoattractants (*CXCL1*, *CXCL2*, *CXCL6*, *CXCL8, CSF3)* under M5‐induced psoriatic conditions. This upregulation was accompanied by NF‐κB activation, a key inflammatory driver that is aberrantly activated in psoriatic skin.^[^ ^45^, ^46^ ^]^ Pharmacological inhibition of NF‐κB reversed this effect, confirming its role as a downstream mediator of FADS2 deficiency. Conversely, *FADS2* overexpression attenuated M5‐induced inflammation, suggesting that FADS2 might mitigate the severity of psoriasis by modulating inflammatory responses in keratinocytes through NF‐κB signaling pathways, limiting excessive cytokine production and immune cell infiltration. Collectively, these findings revealed a novel anti‐inflammatory role of FADS2 in psoriasis by limiting NF‐κB activation and disrupting the keratinocyte‐neutrophil inflammatory loop.

PUFAs synthesized by FADS2 and other enzymes, such as ELOVL5 and FADS1, form an intricate bioactive network that is crucial for maintaining skin homeostasis. PUFAs exert opposing effects on inflammation and are increasingly implicated in the pathogenesis of inflammatory skin disorders, including psoriasis.^[^ ^47^ ^]^ Clinical studies have reported altered circulating PUFA levels in patients with psoriasis and their correlation with psoriasis severity.^[^ ^3^ ^]^ Mechanistically, n‐3 PUFA, such as DHA and EPA, act as endogenous ligands for Toll‐like receptors (TLRs) and suppress pro‐inflammatory signaling mediated by TLR‐2 in human monocytes.^[^ ^48^ ^]^ Additionally, both DHA and EPA inhibit TLR‐4, the receptor responsible for recognizing lipopolysaccharide (LPS) and exert inhibitory effects on LPS‐triggered NF‐κB activation, reducing the production of inflammatory mediators, including cytokines (TNF‐α, IL‐1, IL‐6, IL‐8, and IL‐12), cyclooxygenase‐2, and inducible nitric oxide synthase in various cell types.^[^ ^49^, ^50^ ^]^ N‐3 PUFAs alleviated inflammation in a psoriasis‐like mouse model by inhibiting Th17 responses and promoting Treg cell activation, leading to reduced levels of IL‐17, IL‐22, and IL‐23 and enhanced expression of anti‐inflammatory molecules, such as FOXP3.^[^ ^51^ ^]^ In keratinocytes, n‐3 PUFAs suppress UVB‐induced and cytokine‐driven inflammation, further supporting their anti‐inflammatory role in the epidermis.^[^ ^17^, ^52^, ^53^ ^]^ A recent meta‐analysis confirmed that n‐3 PUFA supplementation significantly improved clinical outcomes in psoriasis, with greater benefits observed at higher doses (>1800 mg day^−1^) and shorter treatment durations (<8 weeks).^[^ ^54^ ^]^ However, whether PUFA biosynthesis is intrinsically dysregulated within psoriatic keratinocytes and how this dysregulation contributes to disease pathophysiology remain unclear. To address this, we employed the desaturation index, which is the ratio of product to precursor fatty acids (e.g., DHA/EPA and DHA/ALA), as a sensitive proxy for FADS2 activity. Compared to absolute fatty acid levels, the desaturation index more accurately reflects FADS2 (Δ6‐desaturase) activity, minimizing confounding effects from upstream substrate accumulation, downstream enzyme compensation, altered lipid mobilization, or other compensatory as well as feedback regulatory mechanisms.^[^ ^7^, ^55^ ^]^ Using this approach, we demonstrated that *FADS2* silencing disrupts DHA biosynthesis in keratinocytes, a key anti‐inflammatory n‐3 PUFA with known efficacy in reducing skin irritation and inflammation when topically delivered via resveratrol‐based nanoparticles.^[^ ^17^ ^]^ Furthermore, DHA supplementation attenuated M5‐induced inflammatory responses and NF‐κB phosphorylation in keratinocytes. Notably, supplementation with DHA partially reversed the heightened expression of inflammatory mediators in FADS2‐deficient keratinocytes, suggesting that restoration of the PUFA balance can mitigate inflammation, even in the context of impaired FADS2 activity. Together, these findings highlight the critical role of FADS2 in inflammatory signaling of keratinocytes by regulating PUFA metabolism and suggest that targeting lipid metabolic reprogramming, specifically via the FADS2‐DHA axis, may represent a promising therapeutic strategy for psoriasis.

The transcriptional regulation of FADS2 is influenced by various physiological and environmental factors, including diet, hormones, and transcription factors such as PPARα and SREBP1.^[^ ^27^, ^33^, ^56^ ^]^ A previous study has shown that PPARα upregulates FADS2 expression by directly binding to its promoter, thereby attenuating dehydroepiandrosterone‐induced ferroptosis in primary granulosa cells.^[^ ^57^ ^]^ Similarly, SREBP1 has been implicated in enhancing FADS2 expression and promoting saponin metabolism.^[^^56]^ In fish, both PPARα and SREBP1 contribute to FADS2 activation, supporting species‐specific differences in FADS2 regulation across various species.^[^ ^27^ ^]^ To clarify the upstream regulatory factors of FADS2 in psoriasis, we first assessed the expression profiles of PPARα and SREBP1. Notably, PPARα expression was decreased in psoriatic lesions, closely paralleling the suppression of FADS2. In contrast, SREBP1 expression was either unchanged or elevated, suggesting that PPARα, rather than SREBP1, is the more plausible upstream regulator of FADS2 under psoriatic conditions. Functional validation in keratinocytes supported this hypothesis: silencing *PPARA* in keratinocytes significantly reduced *FADS2* expression, while activation of PPARα using the selective agonist WY14643 effectively restored FADS2 levels. These results identify PPARα as a critical positive regulator of FADS2 in psoriasis. Beyond its metabolic role, PPARα has well‐documented anti‐inflammatory properties in skin. It modulates keratinocyte proliferation, differentiation, and cytokine responses, thereby contributing to tissue homeostasis and repair.^[^ ^58^ ^]^ Topical application of WY14643 has previously been shown to suppress antigen‐induced cutaneous inflammation in an atopic dermatitis model.^[^ ^59^, ^60^ ^]^ In line with these reports, we found that *PPARA* silencing enhanced M5‐induced pro‐inflammatory gene expression in keratinocytes, whereas PPARα activation dampened this response. Moreover, in the IMQ‐induced psoriasis‐like mouse model, WY14643 treatment significantly ameliorated skin inflammation. To further determine whether the anti‐inflammatory effects of PPARα are mediated through FADS2, we performed loss‐of‐function rescue experiments in both keratinocytes and mouse models. Interestingly, *FADS2* silencing abolished the therapeutic effects of PPARα activation, both in vitro and in vivo. These findings demonstrate that the immunomodulatory effects of PPARα are critically dependent on FADS2 expression.

Our findings revealed a functional PPARα‐FADS2 regulatory axis that governs keratinocyte‐intrinsic inflammatory responses in psoriasis. Activation of PPARα restores FADS2 expression, reestablishes PUFA homeostasis, and suppresses NF‐κB‐mediated cytokine and chemokine production, ultimately inhibiting neutrophil recruitment in cutaneous inflammation. These findings not only highlight a previously underappreciated mechanism of inflammation control in psoriasis but also position PPARα‐FADS2 signaling as a promising therapeutic target. Given the limitations of current biological treatments for psoriasis, including high cost, side effects, and relapse potential, targeting this metabolic‐immunological axis may offer a viable alternative therapeutic approach. Furthermore, the anti‐inflammatory benefits of PPARα agonists observed in other dermatological conditions underscore the broader therapeutic relevance of this pathway.

Although this study provides new insights into the immunometabolic role of keratinocyte‐intrinsic FADS2 in psoriasis, it has several limitations. First, although PPARα was identified as a key transcriptional activator of FADS2, the precise regulatory mechanism remains unclear. Other transcriptional or epigenetic modulators may also be involved, and further investigations using promoter analyses, ChIP‐seq, or CRISPR‐based screens are required to map the regulatory network. Second, the interpretation of PUFA dysregulation, particularly the paradoxical increase in certain fatty acids upon *FADS2* knockdown, may be confounded by compensatory pathways involving other enzymes such as FADS1 or ELOVL5. Although the desaturation index provides some insights, it cannot fully exclude these confounders. Isotope‐labeled tracing studies could help clarify PUFA flux alterations. Finally, although the IMQ‐induced psoriasis model captures key inflammatory features, it does not fully mimic the chronic and relapsing nature of human disease. Validation using alternative models, such as IL‐23 injection or xenografted human skin, would strengthen the translational relevance of our findings. The IL‐23 intradermal injection model more closely recapulates the IL‐23/Th17‐driven immune axis in human psoriasis and has shown strong predictive value for biologic therapies targeting this axis.^[^ ^61^, ^62^ ^]^ Humanized xenograft models further offer an in vivo platform to study human skin inflammation and treatment responses, preserving the structural and immunological features of human skin.^[^ ^63^, ^64^ ^]^ These models may provide useful avenues for future validation of the PPARα–FADS2 signaling pathway in psoriasis.

In conclusion, our study reveals that FADS2 is a critical link between lipid metabolism and keratinocyte‐mediated inflammation in psoriasis. FADS2 downregulation impairs PUFA homeostasis, activates NF‐κB signaling, and promotes neutrophil recruitment. Activation of the PPARα‐FADS2 axis effectively attenuates inflammation in vitro and in vivo, underscoring its therapeutic potential. These findings provide new insights into immunometabolism in psoriasis and highlight lipid metabolic modulation as a promising treatment strategy.

## 4 Experimental Section

### Patients

Skin samples were obtained from patients with moderate‐to‐severe psoriasis (PASI ≥10) and healthy volunteers. These samples were subjected to RNA extraction or paraffin embedding. All procedures for the collection of samples, including skin biopsies, were approved by the Ethics Committees of Shanghai Skin Disease Hospital and Shanghai Tenth People's Hospital and were conducted in accordance with the Declaration of Helsinki. Written informed consent was obtained from all participants prior to sample collection.

### Histologic Analysis and Immunofluorescence Staining

For histological evaluation, skin samples were fixed in 4% paraformaldehyde, paraffin‐embedded, sectioned at a thickness of 4 µm, and stained with hematoxylin and eosin. Images were captured using an Olympus microscope. Epidermal thickness and dermal cell infiltration were quantified in randomly selected regions using ImageJ software (version 1.52a).

For immunohistochemical analysis, skin sections were deparaffinized through a series of xylene and ethanol solutions, followed by antigen retrieval in 1 mM EDTA buffer (pH 9.0) and quenching of endogenous peroxidase with 3% H_2_O_2_. Sections were blocked with 2% bovine serum incubated overnight at 4 °C with primary antibodies (rabbit anti‐FADS2 polyclonal antibody (pAb) (#NBP2‐82734, R&D); mouse anti‐K14 monoclonal antibody (mAb) (#ab7800, Abcam); rabbit anti‐Ki67 mAb (#ab16667, Abcam); rabbit anti‐PPARα mAb (#PA1‐822A, Invitrogen); rabbit anti‐Phospho‐NF‐κB p65 (Ser536) mAb (#3033, Cell Signaling Technology); rabbit anti‐Ly6G mAb (#ab238132, Abcam); rabbit anti‐ELOVL5 pAb (#26599‐1‐AP, Proteintech); rabbit anti‐FADS1 pAb (#10627‐1‐AP, Proteintech)). After washing, a two‐/three‐color fluorescence staining kit (Recordbio Biological Technology) based on tyramide signal amplification was used to stain the sections according to the manufacturer's instructions. The sections were then stained with DAPI (#4083, Cell Signaling Technology). Images were captured using an Olympus fluorescence microscope. Signals from different channels were merged using ImageJ software (version 1.52a).

### Mice and IMQ‐Induced Psoriasis Mouse Model

Wild‐type female C57BL/6 mice and wild‐type female BALB/c mice were purchased from the Shanghai Jihui Laboratory Animal Breeding Co., Ltd. and housed in a specific pathogen‐free facility. All animal procedures were conducted in accordance with national guidelines and approved by the Ethics Committee of Shanghai Skin Disease Hospital and Shanghai Tenth People's Hospital (Shanghai, China). Experiments were conducted when mice were ≈7 weeks old.

To induce psoriasis‐like skin inflammation on the dorsal skin, a daily dose of 62.5 mg of 5% IMQ cream (#H20030128, Mingxin Pharmaceuticals) was topically applied to a shaved 2.5 cm × 2.5 cm area on the back for 6 consecutive days. Control mice received an equal amount of Vaseline. Skin lesions were photographed and scored daily, and mice were sacrificed on day 6 for sample collection.

To induce psoriasis‐like skin inflammation on the mouse ear, 25 mg of 5% IMQ cream was applied to each ear daily for 11 consecutive days. Control mice received an equal amount of vaseline. Mice were sacrificed and the skin samples were collected on day 11.

### Topical siRNA Delivery and PPARα Agonist Treatment

To silence the expression of *Fads2* in mouse ear, 2 nmol *Fads2* siRNA (si*Fads2*) (sense: GCGUUUCUUCUA CACCUACAUTT; antisense: AUGUAGGUGUAGAAGAAACGCT; Hippobiotec) or control siRNA (siNC) were mixed with Lipofectamine 3000 (#L3000015, Invitrogen) and base cream (containing stearic acid, liquid paraffin, white petrolatum, lanolin, and triethanolamine). The mixture was topically applied to the ears every 48 h, and 4 h after IMQ treatment.

To activate PPARα in dorsal skin, 60µl of 10 mm PPARα agonist WY14643 (HY‐16995, Medchemexpress) dissolved in a 7:3 (v/v) mixture of propylene glycol and ethanol (vehicle) was applied topically once daily starting one day before IMQ treatment, and 4 h after IMQ treatment, for 6 consecutive days.

To investigate whether the anti‐inflammatory effect of PPARα was mediated through FADS2, 20 µL of 10 mm WY14643 or vehicle was topically applied to the mouse ears once daily before IMQ treatment. In parallel, 2 nmol of si*Fads2* or siNC formulated with Lipofectamine 3000 and base cream was topically applied to the ears every 48 h, 4 h after IMQ treatment.

### Adeno‐Associated Virus (AAV)‐Mediated Gene Manipulation

To manipulate FADS2 expression specifically in epidermal keratinocytes, 6–week‐old female BALB/c mice were intradermally injected with AAV9 vectors driven by the keratinocyte‐specific keratin 14 (K14) promoter: AAV9‐K14‐sh*Fads2*, AAV9‐K14‐shNC or AAV9‐K14‐flag‐*Fads2*, AAV9‐K14‐Ctrl (titer:2.5 × 10_1__1_; HanBio Technology). For dorsal skin delivery, a 2.5 cm × 2.5 cm shaved region in the center of the back was selected. Five intradermal injection sites were arranged in a grid‐like pattern (50 µL per site, ≈0.5 cm apart, 1–2 mm deep). After injection, sterile cotton balls were applied to prevent viral reflux. For auricular delivery, 20 µL of AAV9‐K14‐sh*Fads2*, or AAV9‐K14‐shNC (titer: 5 × 10_11_ HanBio Technology) was intradermally injected into the center of the ear. Three weeks post‐injection, FADS2 expression was assessed by immunoblotting and immunofluorescence staining. IMQ‐induced psoriasis‐like dermatitis was subsequently established at the injection sites to evaluate inflammatory responses.

### Cell Culture and Treatment

The human‐immortalized keratinocyte cell line HaCaT was cultured in Dulbecco's Modified Eagle's Medium supplemented with 10% fetal bovine serum and 1% penicillin–streptomycin at 37 °C in a humidified atmosphere containing 5% CO_2_.

To silence *FADS2* or *PPARA*, HaCaT cells were transfected with siRNA targeting *FADS2* or *PPARA* or control siRNAs, using Lipofectamine RNAiMAX (#13778030, Invitrogen) according to the manufacturer's protocol. The sequences of siRNA are provided in Table S1 (Supporting Information). After 24 h of transfection, cells were stimulated with a cytokine cocktail (10 ng mL^−1^ M5 concluding IL‐1α (#200‐01A, PeproTech), TNF‐α (#300‐01A, PeproTech), IL‐17A (#200‐17, PeproTech), IL‐22 (#200‐22, PeproTech), Oncostatin M (#300‐10, PeproTech)) for another 12 h. Cells were harvested 36 h post‐transfection for subsequent analysis.

To overexpress *FADS2*, transfection of plasmids was performed using Lipofectamine 2000 (#11668027, Invitrogen) according to the manufacturer's instructions, followed by 10 h M5 stimulation or subsequent analysis 48 h later.

To verify whether *FADS2* silencing promotes inflammation through the NF‐κB signaling pathway, HaCaT cells were transfected with si*FADS2* or siNC for 24 h followed by treatment with the NF‐κB inhibitor BAY 11–7082 (5 µм; #HY‐13453, MedChemExpress) or DMSO for 2 h before M5 stimulation for 12 h.

To investigate the anti‐inflammatory effect of DHA on keratinocyte, HaCaT cells were pretreated with 100 µm DHA (#HY‐B2167, Medchemexpress) or vehicle dissolved in ethanol and bound to fatty acid‐free bovine serum albumin in complete growth medium for 36 h and then stimulated with 10 ng mL^−1^ M5 for another 12 h.

To investigate whether DHA supplementation could reverse the enhanced inflammatory response caused by *FADS2* knockdown, HaCaT cells were transfected with si*FADS2* or siNC for 12 h, followed by treatment with 100 µm DHA for 12 h prior to M5 stimulation for an additional 12 h.

To explore the effect of PPARα on keratinocyte inflammation, HaCaT cells were pretreated with 50 µm WY14643 (HY‐16995, Medchemexpress) or DMSO for 21 h, followed by M5 for 3 h.

To assess whether PPARα modulates inflammation through FADS2 regulation, HaCaT cells were transfected with si*FADS2* or siNC for 24 h, followed by treatment with WY14643 or vehicle for 4 h prior to 12 h M5 stimulation.

### RNA Extraction and Quantitative Real‐Time PCR

Total RNA was extracted from HaCaT cells and human/mouse skin tissues using TRIzol (#9109, TaKaRa) according to the manufacturer's protocols. Total RNA was reverse transcribed to cDNA with PrimeScript RT Master Mix (#6210B, TaKaRa). Quantitative real‐time PCR was performed using TB Green Premix Ex Taq (#RR091A, TaKaRa). The primers were designed and synthesized by Sangon Biotech. The primer sequences used for PCR amplification are provided in Table S2 (Supporting Information).

### Immunoblotting

Total proteins were harvested from HaCaT cells and mouse skin tissues using radioimmunoprecipitation assay lysis buffer (#P0013B, Beyotime) containing protease and phosphatase inhibitors (#78442, Thermo). A Pierce BCA Protein Assay Kit (#23227, Thermo) was applied for the quantification of protein concentration according to the manufacturer's instructions. Equal amounts of protein were separated on 10% SDS‐PAGE gels and transferred onto polyvinylidene fluoride membranes. Membranes were blocked by 5% non‐fat milk for 1 h and incubated overnight at 4 °C with primary antibodies (anti‐Cyclophilin B (#43603, CST), anti‐FADS2 (#NBP2‐82734, R&D), anti‐PPARα (#PA1‐822A, Invitrogen), anti‐Phospho‐NF‐κB p65 (#3033, CST), anti‐NF‐κB p65 (#8242, CST). After washing, membranes were incubated with secondary antibodies for 1 h at room temperature. Protein signals were detected using the Odyssey Imaging System.

### Enzyme‐Linked Immunosorbent Assay (ELISA)

The protein levels of murine CXCL1 (#70‐EK296/2), IL‐17A (#70‐EK217/2), and IL‐1β (#70‐EK201B/4) in mouse psoriatic skin lesions were quantified using ELISA kits (Lianke Bio, China), following the manufacturer's instructions. Similarly, human CXCL1 (#70‐EK196), CSF3 (#70‐EK169), and CXCL8 (#70‐EK108/2) levels in cultured human keratinocyte lysates and supernatants were measured using corresponding human ELISA kits (Lianke Bio, China) according to the provided protocols.

### *RNA* Sequencing

Total RNA was extracted from HaCaT cells utilizing RNAiso Plus (#TaKaRa, #9109). RNA quality and integrity were assessed prior to sequencing. ≈1 µg of total RNA was used to construct a cDNA library with an average insert size of 300 ± 50 bp. This library underwent sequencing on the Illumina Novaseq 6000 platform with 2 × 150 bp paired‐end sequencing chemistry. The expression levels of all transcripts were quantified as fragments per kilobase of transcript per million mapped. DEGs were identified based on a fold change greater than 1.5, with a significance threshold of *P* < 0.05. GO analysis and GSEA of KEGG pathway were conducted to evaluate DEGs between the si*FADS2* and siNC groups following M5 stimulation.

### Skin Cell Preparation and Flow Cytometry

The mouse skin tissues were harvested, subcutaneous fat was removed, and tissues were finely minced into small pieces. Samples were digested in 10 mL of RPMI 1640 Medium (#11875093, Gibco), supplemented with 0.25% collagenase (# C9091, Sigma), 0.001 m sodium pyruvate (# BP356, Thermo), 0.01 m HEPES (# BP310, Thermo), and 0.1 mg mL^−1^ DNase (# DN25, Sigma) for 1–1.5 h at 37 °C under gentle rotation. Digestion was terminated with 1 mL fetal bovine serum. Cell suspensions were passed through a 40 µm filter, centrifuged, and resuspended in FACS buffer (2% FBS in PBS). TruStain FcX antibody was used to treat isolated skin cell suspension to block Fc receptors for 10 min at room temperature. Subsequently, cells were stained for 30 min at 4 °C with fluorescently labeled antibodies targeting surface markers: CD45 for leukocytes; Among CD45^+^ cells, CD3 for T cells, CD11b and Ly6G for neutrophils, CD11b and F4/80 for macrophages, CD11c for dendritic cells. SYTOX Blue Dead Cell Stain (# S34857, Invitrogen) was added for viability assessment. Before flow cytometry analysis, 10 000 counting beads (#C36950, Invitrogen) were added to each sample. Samples were analyzed using a FACSAria Fusion Sorter (BD Biosciences), and data were processed using FlowJo v.10 software (TreeStar).

### Fatty Acid Composition Analysis

Cell suspensions were transferred into 15 mL glass centrifuge tubes, and 25 µL of 1 mg mL^−1^ nonadecanoic acid (Sigma, USA) was added as the internal standard. Lipid extraction was performed by introducing 6 mL of a chloroform‐methanol solution (v/v = 2:1), and rotating at 180 rpm for 30 min at room temperature. After adding 2 mL of H_2_O, samples were vortexed at 1,000 rpm for 2 min and centrifuged at 1000 rpm for 10 min. The lower chloroform‐lipid layer (≈4 mL) was collected and dried under a nitrogen stream. Lipid esterification was then conducted by adding 6 mL of a hexane‐0.4 mol L^−1^ potassium hydroxide‐methanol solution (v/v = 1:1), followed by incubation at 37 °C for 30 min. After the reaction, an additional 2 mL of H_2_O was added, vortexed, and centrifuged. The upper organic phase (≈3 mL) was collected and evaporated under nitrogen, then reconstituted in 200 µL of hexane, followed by vortexing. After centrifugation, the upper hexane layer containing fatty acid methyl esters (FAMEs) and the internal standard was injected into the GC–MS system (7890B‐5977B, Agilent Technologies Inc., USA), equipped with a capillary column (30 m × 0.25 mm × 0.25 µm) (DB‐wax, Agilent Technologies Inc., USA). Quantification was executed following normalization to the internal standard, and fatty acids were identified based on FAMEs (37 Component FAME Mix, CDAA‐252795, ANPEL Laboratory Technologies, Shanghai, China), either by separation on GC‐MS or by matching spectra against the NIST library. Data acquisition and analysis were conducted using Mass Hunter Software (Agilent Technologies Inc., USA).

### Statistical Analysis

Statistical analysis was conducted utilizing GraphPad Prism 9.0 software. The two‐tailed Student's t‐test, one‐way ANOVA, or two‐way ANOVA was applied as appropriate. *P* value < 0.05 was considered statistically significant. Data were presented as mean ± standard deviation (SD).

## Conflict of Interest

The authors declare no conflict of interest.

## Author Contributions

J.C., X.Z., Y.Z., and L.C. contributed equally to this work. C.Y.G., Y.L.S., and J.J.L. conceived the study; J.C. performed the experiments; X.Z., Z.Y. performed experiments on clinical patient samples; J.C., X.Z., Y.Z., and L.C. assisted in animal experiments; R.M., Y.C. and N.Y., and Q.C. participated in immunostaining and imaging; Y.W., P.Z., L.Y., X.Z., and Y.H. supported data analysis; X.S., Q.Y. provided support for qPCR experiments; C.Y.G. helped in experiment design. J.C., X.Z., Y.Z., and L.C. interpreted the data and wrote the manuscript.

## Supporting information



Supporting Information

## Data Availability

The data that support the findings of this study are available from the corresponding author upon reasonable request.
