# Supplementary material for: Reprogramming of Fatty Acid Metabolism via PPARα‐Orchestrated FADS2 in Keratinocytes Modulates Skin Inflammation in Psoriasis
Source: Adv Sci (Weinh). 2025 Aug 29;12(40):e17049. doi: 10.1002/advs.202417049 (PMC12561275; doi:10.1002/advs.202417049)
Supplement: Supplementary file 1 — Supporting Information [file ADVS-12-e17049-s001.docx]

Supporting information

**Reprogramming of Fatty Acid Metabolism via PPARα-orchestrated FADS2 in Keratinocytes Modulates Skin Inflammation in Psoriasis**

*Jiangluyi Cai^#^, Xue Zhou^#^, Yu Zhuang^#^, Lian Cui^#^, Rui Ma, Youdong Chen, Nan Yang, Qianyu Chen, Yuanyuan Wang, Peiyao Zhu, Lingling Yao, Qian Yu, Xiaomin She, Xuyang Zhou, Yuemeng Huang, Zengyang Yu, Xilin Zhang, Jiajing Lu*, Yuling Shi*, Chunyuan Guo**


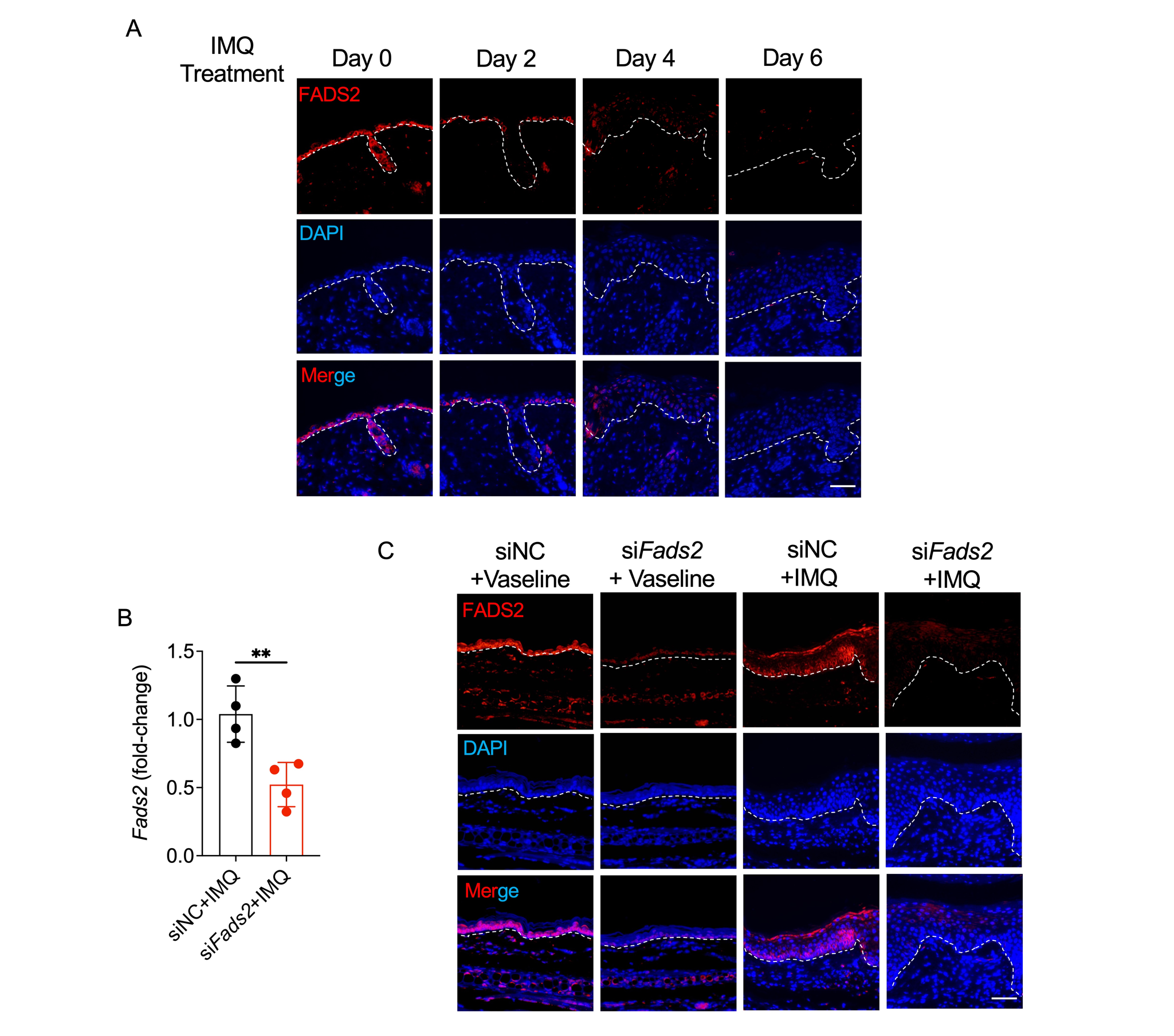


**Supplementary Figure S1.** FADS2 expression in skin lesions during IMQ-induced psoriasis-like mouse model and following topical application of *Fads2* siRNA.

(A) Representative immunofluorescence images of FADS2 in skin lesions from IMQ-induced psoriasis-like mouse model at indicated time points. (B) RT-qPCR analysis of *Fads2* in mouse ear skin lesions after 11 days of IMQ treatment with topical application of *Fads2* siRNA (si*Fads2*) and control siRNA (siNC) (n=4). (C) Representative immunofluorescence images of FADS2 in mouse ear skin lesions treated with si*Fads2* and siNC after 11-day application of Vaseline or IMQ. Scale bar, 50 μm. Data are presented as mean ± SD. Statistical significance was determined by paired two-tailed Student’s t test. ***P* < 0.01.


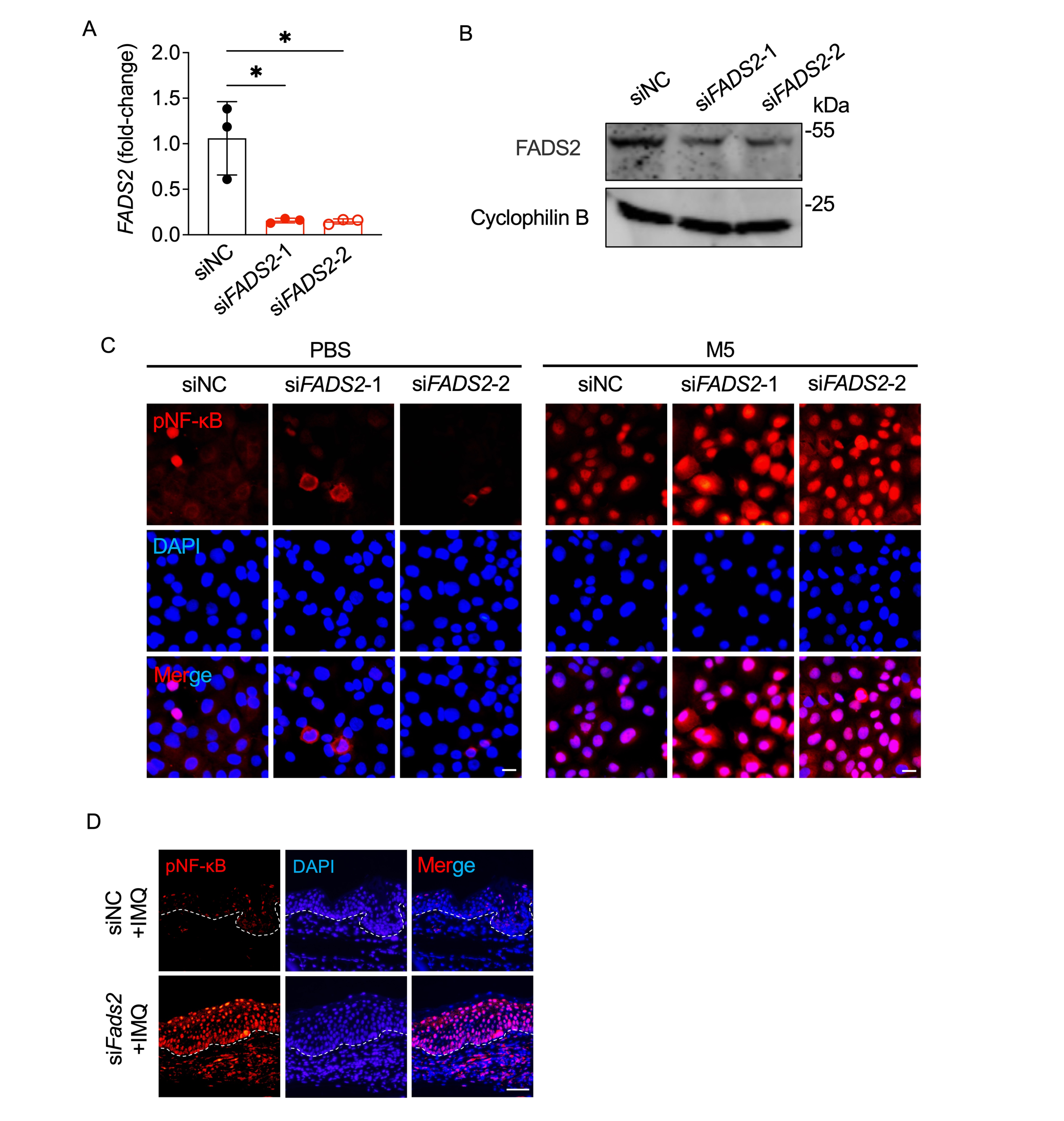


**Supplementary Figure S2.** FADS2 knockdown increases phosphorylated NF-κB levels in keratinocytes of both in vitro and in vivo psoriatic models.

(A and B) RT-qPCR analysis of *FADS2* mRNA expression (A) and immunoblotting of FADS2 protein levels (B) in HaCaT cells transfected with si*FADS2* and siNC for 24 hours (n=3). (C) Representative immunofluorescence images of phosphorylated NF-κB p65 (pNF-κB) staining in HaCaT cells transfected with si*FADS2* and siNC for 24 hours, followed by PBS or M5 for 1 hour. (D) Representative immunofluorescence images of pNF-κB staining in mouse ear skin lesions treated with si*Fads2* and siNC after 11 days of IMQ treatment. Scale bar, 50 μm. Data are presented as mean ± SD. Statistical significance was determined by one-way ANOVA. **P* < 0.05.


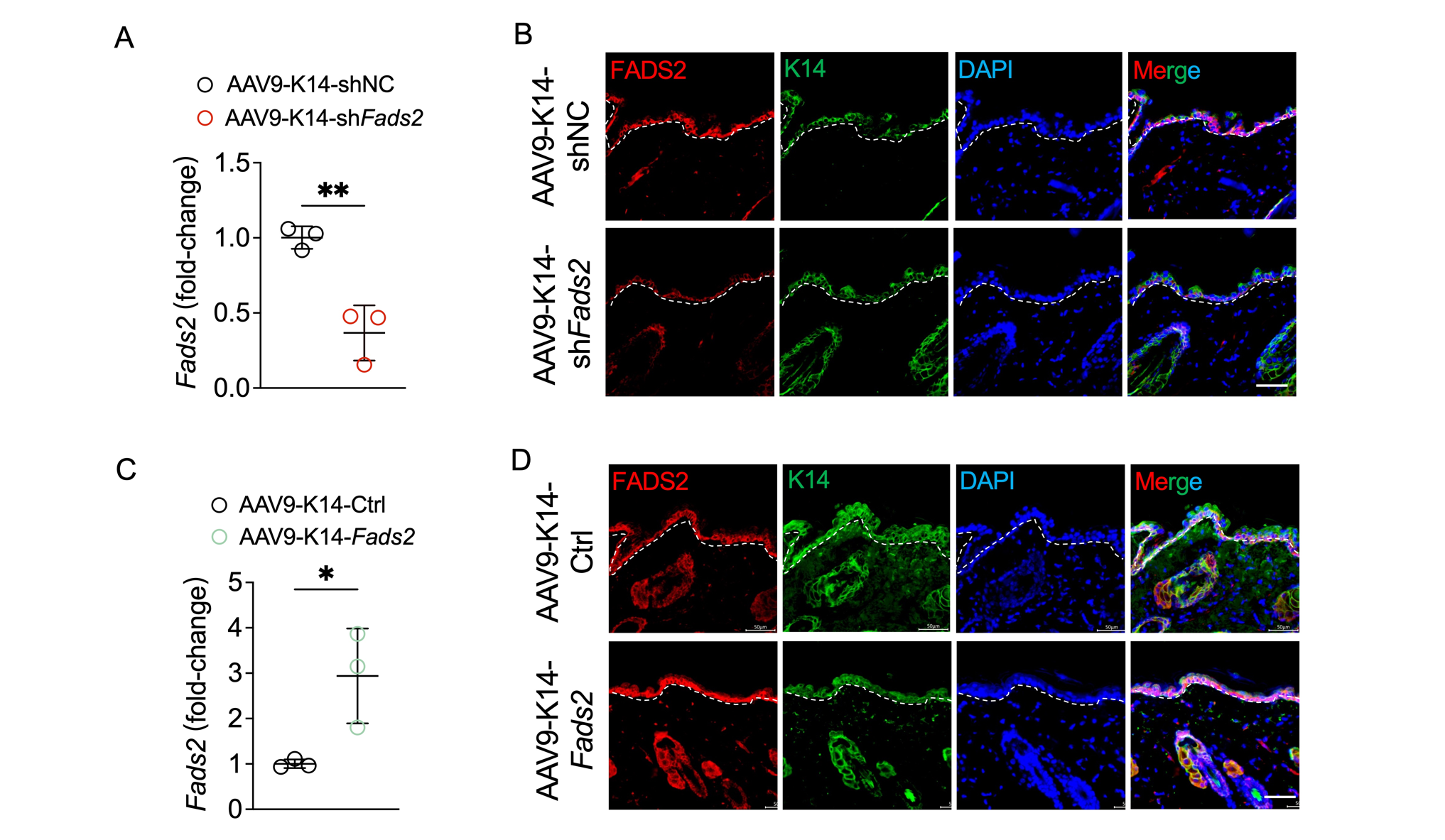


**Supplementary Figure S3.** FADS2 expression is successfully manipulated in epidermal keratinocytes using a K14 promoter-driven AAV9 delivery system in mice.

(A) RT-qPCR analysis of *Fads2* in the epidermis of mouse dorsal skin following intradermal injection of AAV9-K14-sh*Fads2* or AAV9-K14-shNC (n=3). (B) Representative immunofluorescence images of FADS2 and K14 co-staining in dorsal skin from mice treated with AAV9-K14-sh*Fads2* or AAV9-K14-shNC. (C) RT-qPCR analysis of *Fads2* in the epidermis of mouse dorsal skin treated with AAV9-K14-*Fads2* or AAV9-K14-Ctrl (n=3). (D) Representative immunofluorescence images of FADS2 and K14 co-staining in mouse dorsal skin treated with AAV9-K14-Flag- *Fads2* or AAV9-K14-Ctrl (n=3). Scale bar, 50 μm. Data are presented as mean ± SD. Statistical significance was determined by unpaired two-tailed Student’s t test. **P* < 0.05, ***P* < 0.01.


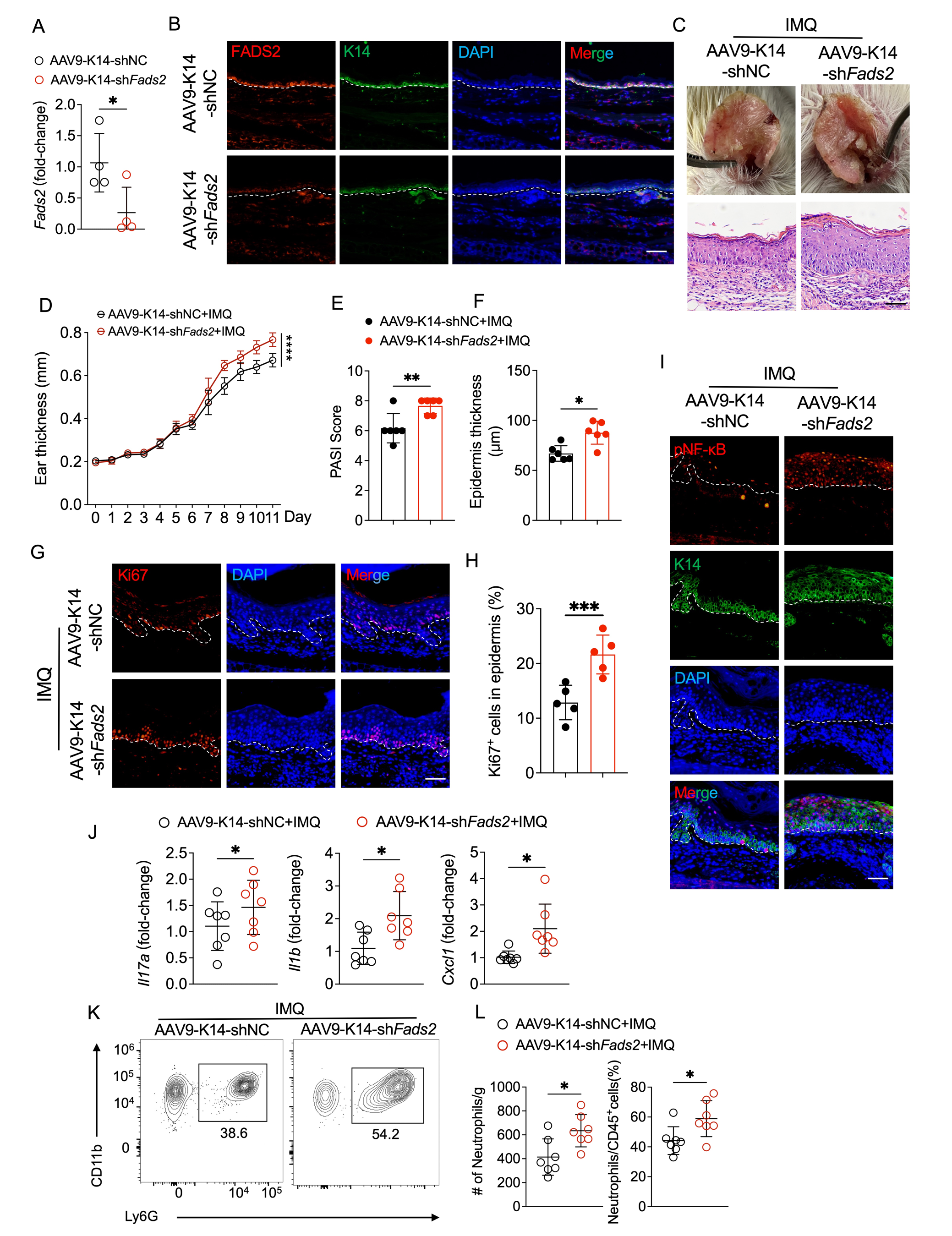


**Supplementary Figure S4.** Keratinocyte-specific knockdown of *Fads2* in the ear exacerbates IMQ-induced psoriasiform dermatitis.

(A) RT-qPCR analysis of *Fads2* in the epidermis of mouse ear skin treated with AAV9-K14-sh*Fads2* or AAV9-K14-shNC (n=4). (B) Representative immunofluorescence images of FADS2 and K14 co-staining in mouse ear skin treated with AAV9-K14-sh*Fads2* or AAV9-K14-shNC. (C and D) Representative phenotypic images and H&E staining images (C), ear thickness of the indicated time points (D), in mice treated with AAV9-K14-sh*Fads2* or AAV9-K14-shNC after IMQ treatment for 11 days (n=7). (E and F) Quantification of PASI scores (E) and epidermal thickness (F) of mouse ear treated with AAV9-K14-sh*Fads2* or AAV9-K14-shNC followed by 11-day IMQ application (n=6). (G and H) Representative immunofluorescence images of Ki67 staining (G) and quantitation of Ki67+ epidermal cells (H) in IMQ-induced skin lesions treated with AAV9-K14-sh*Fads2* or AAV9-K14-shNC (n=5). (I) Representative immunofluorescence images of pNF-κB and K14 co-staining in IMQ-induced skin lesions treated with AAV9-K14-sh*Fads2* or AAV9-K14-shNC. (J) RT-qPCR analysis of the indicated genes in IMQ-induced skin lesions treated with AAV9-K14-sh*Fads2* or AAV9-K14-shNC (n=7). (K and L) Representative flow cytometry plot (K) and quantification (L) of neutrophils in IMQ-induced skin lesions treated with AAV9-K14-sh*Fads2* or AAV9-K14-shNC (n=7). Scale bar, 50 μm. Data are presented as mean ± SD. Statistical significance was determined by paired two-tailed Student’s t test (A,E,F,H,J,L) or two-way ANOVA (D). **P* < 0.05, ***P* < 0.01, ****P* < 0.001, *****P* < 0.0001; ns, not significant


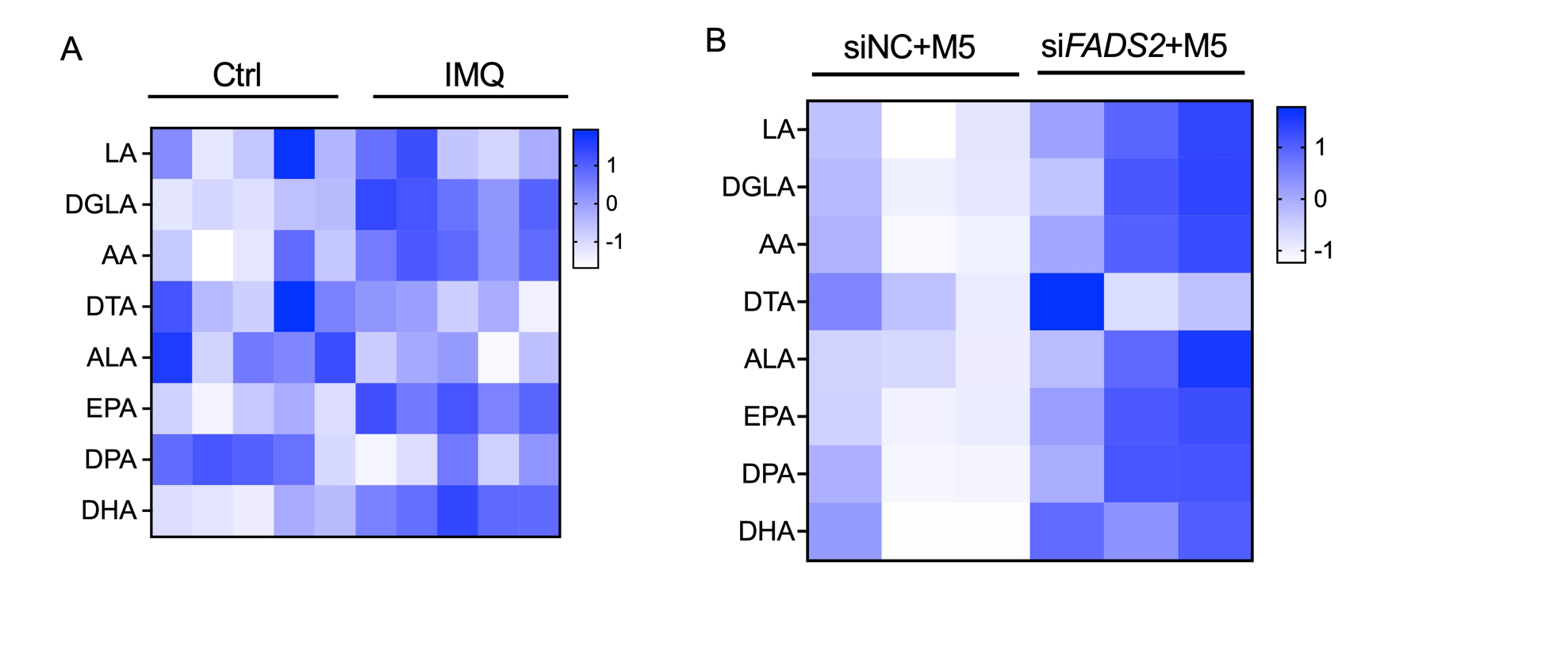


**Supplementary Figure S5.** Lipidomic analysis of PUFAs in the epidermis of IMQ-induced skin lesions and *FADS2*-silenced keratinocytes under M5 stimulation.

(A) Heatmap of intercellular polyunsaturated fatty acid (PUFA) levels in the epidermis of IMQ-induced skin lesions and skin tissue from control mice, assessed by LC-MS/MS (n=4). (B) Heatmap of intercellular PUFA levels in HaCaT cells transfected with si*FADS2* or siNC after M5 stimulation, analyzed by LC-MS/MS (n=3). Data are visualized in the form of a heatmap.


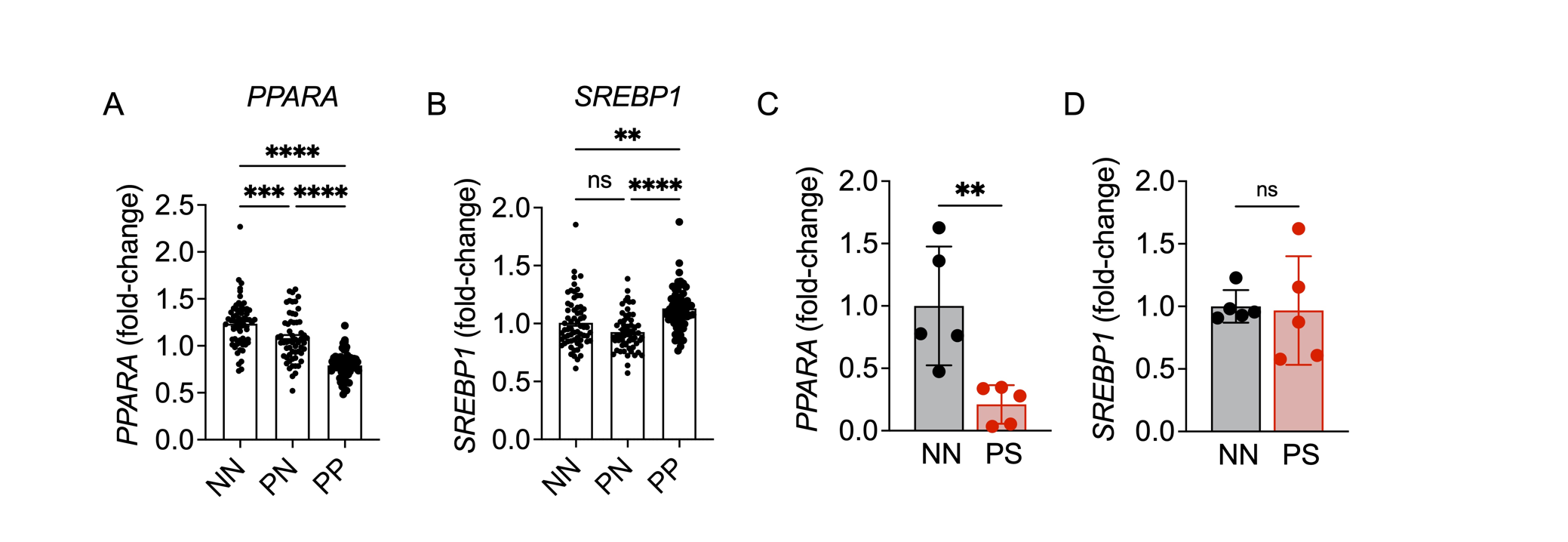


**Supplementary Figure S6.** The expression levels of PPARα and SREBP1 in psoriatic skin lesions.

(A and B) Expression profiling of *PPARA* (A) and *SREBP1* (B) mRNA from lesional (PP) and non-lesional skin (PN) from psoriasis patients (n=58), and normal skin (NN) from healthy controls (n=64), based on public microarray dataset (GSE13355). (C and D) Expression levels of *PPARA* (C) and *SREBP1* (D) in lesional skin from psoriasis patients (PS) (n=5) and normal skin from healthy controls (NN) (n=5), based on published RNA-sequencing data from Yu et al, 2019. Data are presented as mean ± SD. Statistical significance was determined by one-way ANOVA (A,B) or unpaired two-tailed Student’s t test (C,D). ***P* < 0.01, ****P* < 0.001, *****P* < 0.0001; ns, not significant.


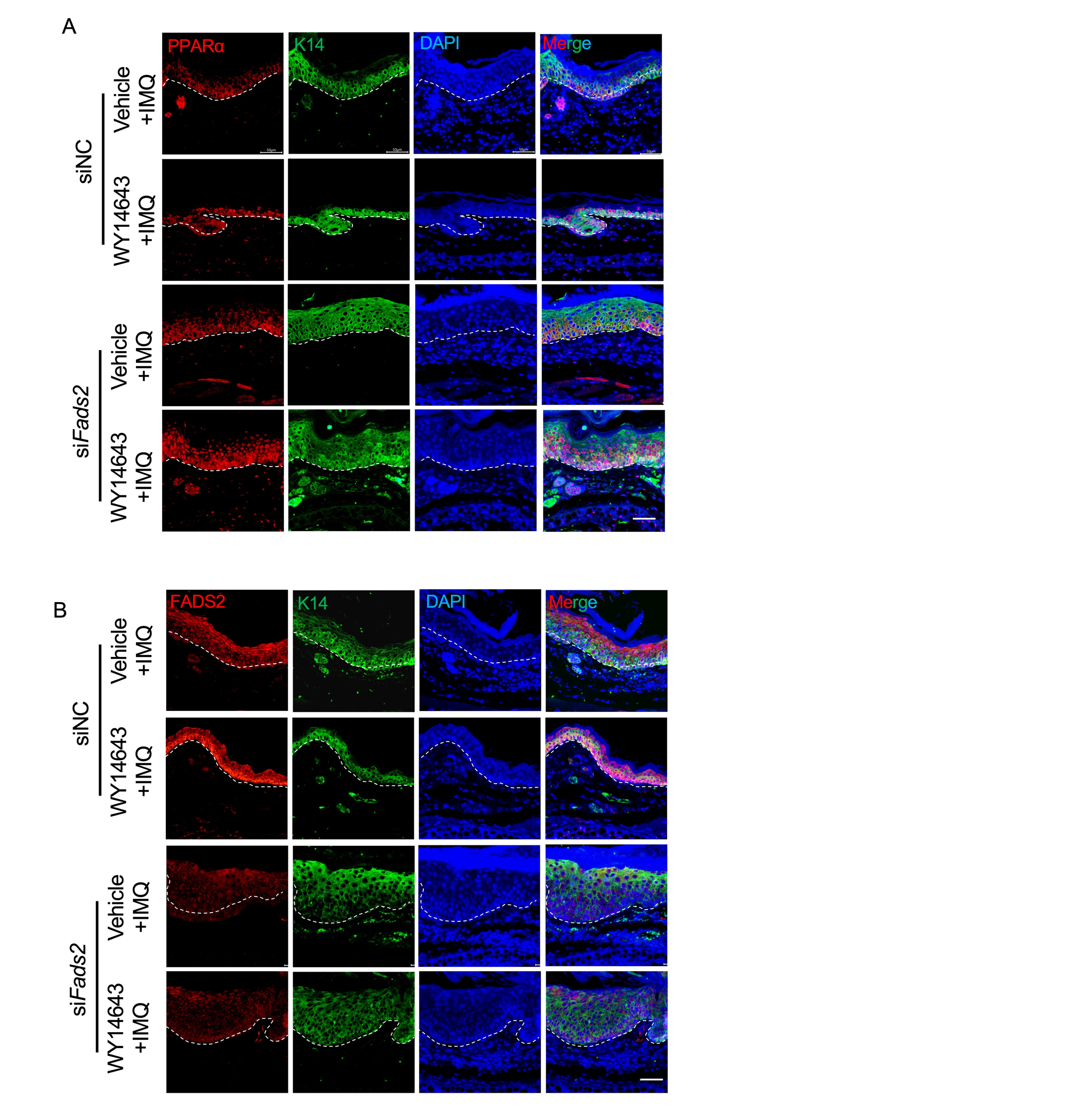


**Supplementary Figure S7.** The expression levels of PPARα and FADS2 in IMQ-induced skin lesions following WY14643 treatment and *Fads2* knockdown.

(A and B) Representative immunofluorescence images of PPARα and K14 co-staining (A), and FASD2 and K14 co-staining (B) in IMQ-induced skin lesions treated with WY14643 or vehicle prior to the application of si*Fads2* and siNC. Scale bar, 50 μm.

# Supplementary Table

Table S1. Sequences of siRNAs

| Target | siRNA ID | Sense Strand (5'→3') | Antisense Strand (5'→3') |
| --- | --- | --- | --- |
| hFADS2 | siRNA-1 | GCGUUUCUUCUACACCUACAUTT | AUGUAGGUGUAGAAGAAACGCTT |
| hFADS2 | siRNA-2 | CGUAAUGUUUAUCAUGUUACU | UAACAUGAUAAACAUUACGAU |
| hControl | siNC | UUCUCCGAACGUGUCACGUTT | ACGUGACACGUUCGGAGAATT |
| hPPARα | siRNA-1 | GCUUUGGCUUUACGGAAUATT | UAUUCCGUAAAGCCAAAGCTT |
| hPPARα | siRNA-2 | GCGUAUGGAAAUGGGUUUATT | UAAACCCAUUUCCAUACGCTA |
| mFads2 | siRNA | GCGUUUCUUCUACACCUACAUTT | AUGUAGGUGUAGAAGAAACGCT |

Table S2. Primer sequence of Quantitative PCR

| Gene Name | Species | Forward Sequence (5'→3') | Reverse Sequence (5'→3') |
| --- | --- | --- | --- |
| *FADS2* | human | AGGATGCCTCGTCTTCCTT | GGCTGCGGACCTCTACCATTA |
| *FADS1* | human | CCAACGTGCTTCCGCAAAGAC | GGTCGTGTGTTGACCGCATA |
| *ELOVL5* | human | AGTGGTGTGATACCTTGGACTCA | ACGAGAGGACACGGATAATCTTC |
| *PPARA* | human | TTGCGAATCCATGCGCGAG | CCACAGGATAAGTCACCGGAG |
| *CXCL1* | human | CAATCCTGCACCCCAATAGT | GGATTTCACGTGTACGCACTTT |
| *CXCL2* | human | AGATCAATGTGACCGCAGGG | TCTCTGCTCTAACACAGAGGGA |
| *CXCL3* | human | AAAGGATACTGAACAGGGGAGCA | CTCTGGTAAGGGGCAGGGACC |
| *CXCL5* | human | AGCTGCGTTGCGTTTGTTTAC | TGGCGGAACACTTCGAGATTAC |
| *CXCL6* | human | AGAGCTGGGTTGCACCTTTT | GGACTTTTACCAATCGTTTTGGGG |
| *CXCL8* | human | AACTGAGAGTGATTGAGAGTGG | ATGAATTCTCAGCCCTCTTCAA |
| *CSF3* | human | GCTGCTTGAGCCAACTCCATA | GAACGCGGTACGGACACCTC |
| *S100A7* | human | GCTGACGATGATGAAGGAGAAGACT | GCGAGGTTAATTTGTGCCCTTT |
| *S100A8* | human | ACGTCTACCAAAGTACTCCGTGAT | TGAGGACACTCGGTCTCTAGCA |
| *S100A9* | human | CAACACCTTCCACCAATACTCTGT | TCGCACCAGCTCTTTGGAATTC |
| *Il17a* | mouse | TTTAACTCCCTTGGGCAAAA | CTTTCCCTCCGCATTGACAC |
| *Tnfa* | mouse | CTGAACCCCGAGTGACAAGC | GGCTTGTCACTCGAATTTTGAGA |
| *Il1b* | mouse | GCAACTGTTCCTGAACTCAACT | ATCTTTTGGGGTCCGTCAACT |
| *Il6* | mouse | TAGTCCTTCCTACCCCAATTTCC | TTGGTCCTTAGCCACTCCTTC |
| *Cxcl1* | mouse | CTGGGATTCACCTCAAGAACATC | CAGGGTCAAGGCAAGCCTC |
| *Cxcl2* | mouse | CCAACCACCAGGCTACAGG | GGCTCACACTCAAGCTCTG |
| *Cxcl3* | mouse | CCAGAGCAGAGACCTTATCCAC | CTTCATCATGGTGAGGGGTT |
| *Cxcl15* | mouse | TGTTGAGCATGAAAGGCCTTAT | AGGTCTCCGGAATTGGAAGG |
| *Csf3* | mouse | ATGGCTCACTTTTGCCTCCAG | CTGACAGTGACCAGGGGAAC |
| *S100a8* | mouse | AAATCACCATGCCCTCTACAAG | CCCACTTTTATCACCATCGCAA |
| *S100a9* | mouse | ATACCTCAGGAAGGAGGAACCC | TCCATGATGTCATTTATGAGGGC |
| *Fads2* | mouse | AAGGGAGGGTAAACGGGAGAGC | CGGCTGGGACCATTTGTGTAA |
